# Simulations Meet Experiment to Reveal New Insights into DNA Intrinsic Mechanics

**DOI:** 10.1371/journal.pcbi.1004631

**Published:** 2015-12-10

**Authors:** Akli Ben Imeddourene, Ahmad Elbahnsi, Marc Guéroult, Christophe Oguey, Nicolas Foloppe, Brigitte Hartmann

**Affiliations:** 1 LBPA, CNRS, ENS Cachan, Université Paris-Saclay, Cachan, France; 2 Université Pierre et Marie Curie, Paris, France; 3 LPTM, UMR 8089, Université de Cergy-Pontoise, Cergy-Pontoise, France; 4 UMR S665, INSERM, Université Paris Diderot, INTS, Paris, France; 5 Cambridge, United Kingdom; Baltimore, UNITED STATES

## Abstract

The accurate prediction of the structure and dynamics of DNA remains a major challenge in computational biology due to the dearth of precise experimental information on DNA free in solution and limitations in the DNA force-fields underpinning the simulations. A new generation of force-fields has been developed to better represent the sequence-dependent B-DNA intrinsic mechanics, in particular with respect to the BI ↔ BII backbone equilibrium, which is essential to understand the B-DNA properties. Here, the performance of MD simulations with the newly updated force-fields Parmbsc0_εζOLI_ and CHARMM36 was tested against a large ensemble of recent NMR data collected on four DNA dodecamers involved in nucleosome positioning. We find impressive progress towards a coherent, realistic representation of B-DNA in solution, despite residual shortcomings. This improved representation allows new and deeper interpretation of the experimental observables, including regarding the behavior of facing phosphate groups in complementary dinucleotides, and their modulation by the sequence. It also provides the opportunity to extensively revisit and refine the coupling between backbone states and inter base pair parameters, which emerges as a common theme across all the complementary dinucleotides. In sum, the global agreement between simulations and experiment reveals new aspects of intrinsic DNA mechanics, a key component of DNA-protein recognition.

## Author Summary

The ability to simulate computationally the structure and dynamics of biomolecules is a major goal of structural biology. Such simulations require the calculation of the forces and energy of the system, typically with extensively parametrized functions called “force-fields”. Developing reliable force-fields has been very challenging for DNA, mainly because the simulations have to reproduce subtle, complex, sequence-dependent differences that also remain difficult to capture experimentally in solution. Here, we take advantage of an extensive set of recent experimental (NMR) data gathered on selected DNA oligomers to test the performance of a new generation of force-fields for DNA simulations. Our results demonstrate impressive progress towards more realistic simulations of DNA. The agreement between experiment and simulations is now good enough to incite further interpretation of the experimental observables and yield new original insights into the intrinsic DNA mechanics. In sum, this work shows that reliable DNA simulations provide a much finer understanding of the structural and dynamical B-DNA behavior, which will be essential to account for DNA recognition by proteins.

## Introduction

Binding of DNA to proteins or small molecules is modulated by subtle sequence-dependent variations inherent to the structure and dynamics of free DNA, which facilitate or disfavor the structural fit with cognate partners [1–4]. Given the many DNA targets, a purely experimental characterization of their structure and dynamics is an enormous task. The structural biology of DNA would be greatly helped if one could describe and predict the sequence-dependent intrinsic mechanical and structural preferences of the double helix. That would pave the way to a fuller understanding of DNA malleability in direct and indirect readout.

Molecular Dynamics (MD) simulations in explicit solvent can potentially explore the properties of any B-DNA sequence of moderate length, considering the extensive sampling afforded by modern computational resources [5, 6]. However, MD simulations are only as reliable as the underlying energy model, typically treated with a classical force-field. Development of force-fields is complex, requires extensive efforts, and needs precise reference experimental data [7]. This latter requirement has been a complicating factor for DNA, given the paucity of reliable experimental data reflecting the fine structural details of DNA in solution [8–10]. The situation has improved in recent years, especially with respect to the DNA backbone, for which additional experimental information have been gathered from X-ray crystallography and NMR (see below). In response, force-field shortcomings regarding the DNA backbone were addressed, including *via* QM studies on model compounds [11–13], motivated by the realization that the backbone is an essential component of the intrinsic mechanical couplings in DNA.

Statistical analyses of X-ray structures of free B-DNA have unveiled that, among the five dihedral angles along the phosphate linkage, ε and ζ present bi-modal distributions [14–19], referred to as BI with ε/ζ:*trans/g-* and BII with ε/ζ:*g-/trans* [20, 21]. In contrast, α, β and γ appear to prefer overwhelmingly one conformation (α/β/γ:*g-/t/g+*) [14, 15, 17, 19]. Importantly, the BI and BII conformers are associated to distinctive values of the helicoidal parameters, X-disp (base displacement), slide, roll and twist [15, 16]. In addition, the density of BI or BII phosphate groups in a window of 4 consecutive base pairs is coupled to the groove dimensions [22]. Hence, the modulation of B-DNA shape observed in X-ray structures is associated with the conformation of ε/ζ backbone dihedrals.

NMR solution studies echoed these findings and provided additional information about the sequence dependent behavior of the BI and BII populations. In NMR, this equilibrium is reflected by the ^31^P chemical shifts (δPs) [21, 23], which can be translated in terms of BII percentages [24, 25]. Correlations between NMR-measured δPs and internucleotide distances [24, 26] reflect the coupling between the backbone states and inter base pair parameters observed in X-ray structures. Consistency between NMR and X-ray results extends to the relation between the BII densities and the width of the minor groove, which can also be observed by NMR [27].

In addition, the compilation of a sizeable set of δPs documented the effect of B-DNA sequence on BII propensities [28], initially inferred from X-ray structures [15, 16]. Of the 10 unique complementary dinucleotides (NpN•NpN), CpG•CpG, CpA•TpG, GpC•GpC, GpG•CpC are characterized by BII percentages markedly higher than the average (21%); ApN•NpT (N: any base) and TpA•TpA can be globally considered as BI-rich; GpA•TpC is an intermediate case, with BII percentage only slightly lower than average. These intrinsic sequence-specific BII propensities in solution were summarized on a scale called TRX [28], by reference to the couplings with Twist, Roll and X-disp. TRX was recently validated further by a large set of δPs collected on new DNA oligomers [27].

This detailed information on DNA in solution offers a precious framework for testing and refining DNA force fields. Thus, the AMBER force fields Parm98 [29] and Parm99 [30] stabilized artefactual α/γ conformations that caused severe distortions in DNA [19, 31]. Such undesirable α/γ transitions were corrected in the subsequent potential Parmbsc0 [12]. First observed by NMR [32, 33] and then generalized and quantified in the TRX scale [34], the modulation of CpG BII propensity by the 3'- and 5'-neighbors was qualitatively retrieved by Parmbsc0 [35]. Also, the sensitivity of the BI ↔ BII equilibrium to the type of monovalent cation (K^+^, Na^+^) was demonstrated by NMR [26]. Parmbsc0 simulations do not seem to reproduce this dependence, yet they suggest a mechanism that could explain how K^+^ and Na^+^ affect the backbone motions [35]. Concerning the CHARMM family of force-fields, the early thorough systematic calibration of the DNA backbone torsional energetics for CHARMM27 [17, 36] prevented artefactual α/γ transitions and resulted in a force-field which treats B-DNA robustly [6, 37, 38]. Importantly, CHARMM27, like Parmbsc0, correctly represent the mechanical coupling between the backbone states and the helical parameters [9, 38, 39].

Nevertheless, the remaining shortcomings in Parmbsc0 MDs [5, 35, 40, 41] cannot be ignored, in particular regarding CpG, CpA and TpG that show a systematic deficit in BII with respect to the NMR data [27, 28, 32–34, 42–45]. The CHARMM27 force-field also did not reproduce the experimentally documented BII percentages [9, 39]. A simulation of the Drew-Dickerson dodecamer with Parmbsc0 [40] and a NMR/modeling study with CHARMM27 [46] also raised the issue of unrealistic BII propensities.

In response, two force-fields were recently conceived to improve the DNA backbone representation: Parmbsc0_εζOLI_ [13], derived from Parmbsc0 -, and CHARMM36 [11], built on CHARMM27. Parmbsc0_εζOLI_ and CHARMM36 were developed guided by DNA X-ray structures and a small set of BII percentages extracted from NMR. In initial tests with B-DNA, both force fields notably increased the sampling of the BII form compared to prior potentials [11, 13]. Since twist and groove shape are coupled to the BI ↔ BII equilibrium, the structural outcome obtained with Parmbsc0_εζOLI_ significantly differs from that yielded by Parmbsc0 [13]. These initial tests are encouraging and call for a more systematic examination of the performance of these potentials, especially in the light of experimental data not used to train the force-fields.

The present work exploits a wealth of recent ^31^P NMR chemical shifts on the DNA backbone motions, to thoroughly evaluate the performance of the Parmbsc0_εζOLI_ and CHARMM36 potentials. These data were collected on four DNA dodecamers [27], independent of those used to develop those force-fields. Together, the dodecamers cover a 39 bp segment in the 5’ half of sequence 601, the best artificial sequence at forming nucleosome core particle [47], which is therefore important to understand how DNA is packaged. The TRX approach [28] combined with the analysis of the ^31^P chemical shifts of the four dodecamers [27] provides evidence that the intrinsic structural characteristics of the free sequence 601 largely account for its strong affinity for the histone core. In addition to their biological relevance, the 72 dinucleotides (NpN) of the four dodecamers behave as expected from the TRX scale regarding the effect of sequence on the BII propensities [27]. These dodecamers and the attending experimental information are therefore ideally suited to evaluate Parmbsc0_εζOLI_ and CHARMM36, with emphasis on the representation of the BI ↔ BII equilibrium and the coupled helicoidal parameters. Importantly, we show how the improvements brought by these new potentials lead to new insights into DNA structure and dynamics, which are essentially consistent across the two force-fields.

The first step was to compare the BII percentages inferred from δP measurements to those generated by MDs. The simulated fine modulation by the sequence is not yet fully satisfactory. For instance the simulated BII populations of some steps, as those of GpC with both force fields, tend to be underestimated. However, Parmbsc0_εζOLI_ and CHARMM36 represent the backbone behavior much more realistically than previous force fields. CHARMM36 in particular shows a very good ability to obtain BII-rich steps. This advance enabled to examine for the first time the conformational combinations corresponding to the states of facing phosphate groups, *i*.*e*. BI•BI, BI•BII, BII•BI and BII•BII. We find that the conformational states of the two facing phosphate groups of any complementary dinucleotide are not correlated in either Parmbsc0_εζOLI_ or CHARMM36 simulations. An important practical consequence is that the populations of the combinations of facing phosphates can be easily deduced from the overall individual BII populations inferred experimentally for every phosphate. This approach reveals that the four dodecamers contain a sizable number of steps where BII-containing states, BI•BII, BII•BI and BII•BII, dominate. Such quantification is critical for accurately describing the conformational landscape explored by the complementary dinucleotides, because the backbone combinations are tightly associated with helical parameters, as documented here. Overall, our results deepen our understanding of the intrinsic B-DNA mechanics, which is a key player in the indirect readout of DNA sequences by proteins.

## Results

### Overview of the simulations

Each of the four dodecamers (Table 1) was simulated with the Parmbsc0_εζOLI_ [13] (P-MDs) or CHARMM36 [11] (C-MDs) force-field, resulting in a total of 8 MDs. The MDs of Oligo 1, 2 and 3 were 450ns each, while for Oligo 4 the trajectories were extended to one microsecond. Additional sampling was performed on Oligo 4 since its alternation of BI and BII-rich dinucleotides is especially relevant to test the convergence of backbone dynamics.

**Table 1:** Sequences of the four studied DNA dodecamers, constituents of the sequence 601.

Oligo 1	5'-TCGTAGCAAGCT-3'•5'-AGCTTGCTACGA-3'
Oligo 2	5'-GCTCTAGCACCG-3'•5'-CGGTGCTAGAGC-3'
Oligo 3	5'-CCGCTTAAACGC-3'•5'-GCGTTTAAGCGG-3'
Oligo 4	5'-CGCACGTACGCG-3'•5'-CGCGTACGTGCG-3'

Discarding the terminal bases, these four dodecamers contain 72 dinucleotides (NpN) that correspond to 36 complementary dinucleotides (NpN•NpN).

**Fig 1:**
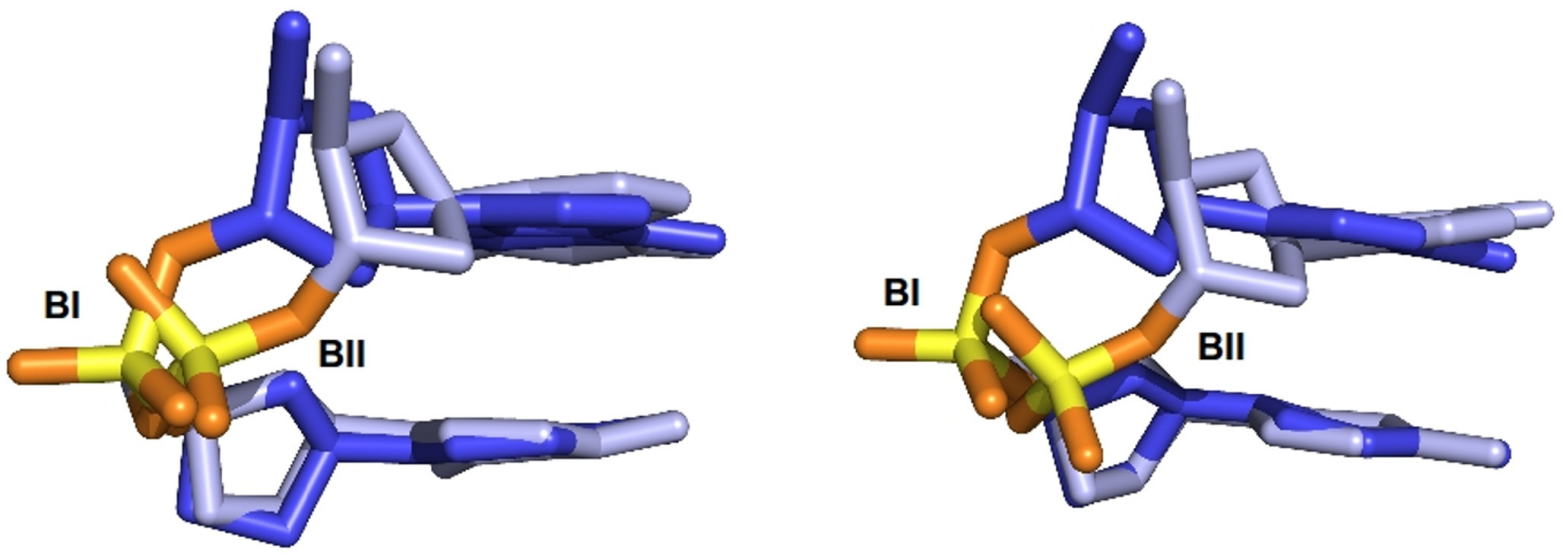
BI and BII conformations in the B-DNA backbone. Illustration of the BI ((ε-ζ) = -90°) and BII ((ε-ζ) = +100°) phosphate linkage conformations with a GpC dinucleotide extracted from MDs carried out with the Parmbsc0_εζOLI_ (left) or CHARMM36 (right) force fields.

During the present simulations, the base pairs N_2_→N_11_•N_14_→N_23_ were stable, with ~99% of Watson-Crick pairing. The root mean square deviations (RMSDs) between a regular canonical B-DNA and the simulated snapshots fluctuated around 2.6±0.6 Å in P-MDs and 2.1±0.5 Å in C-MDs (S1 Fig). The slightly larger RMSDs for P-MDs versus C-MDs gave the first indication of subtle differences between the force-fields, but one should refrain from interpreting these differences in terms of relative validity of the two force-fields, since canonical B-DNA is a somewhat artificial construct. Then, we examined the five dihedral angles of the phosphodiester backbone, α, β, γ, ε and ζ. In both P- and C-MDs, α/β/γ conform to the canonical *g-/trans/g+* pattern observed in free DNA [14, 15, 17–19]. The torsions ε and ζ, which undergo correlated motions, define the BI and BII states (Fig 1). The convergence of the BII populations is of evident relevance, especially to compare simulated BII percentages to their experimental counterpart. Previous analyses of very long trajectories (up to ~45 μs) with Parmbsc0 and CHARMM36 showed reasonable convergence of the fast motions (timescale < 100ns) on internal parts of DNA oligomers after only ~50ns [5]. A similar conclusion was drawn from μs simulations with Parmbsc0, using as convergence criteria the average helical and backbone parameters [41]. These previous studies indicate that the timescale of the present MDs should be amply sufficient to investigate the backbone motions. Indeed, our results confirm this expectation, keeping in mind that constraints were applied to the terminal base-pairs to maintain their Watson-Crick base-pairing. A detailed justification of the protocol is given in Materials and Methods.

Clearly, the BII population of some phosphates did not converge over the first 50 ns, which may be considered as a reasonable equilibration time. Thus, for each phosphate, convergence was monitored by plotting its BII percentage over increasing trajectory lengths, from 150ns upwards (S2 Fig). For each Oligo treated with Parmbsc0_εζOLI_ or CHARMM36, convergence of the BII populations was reached within the simulation times, including for phosphates with high BII populations (S2 Fig).

An additional test was performed with the MDs of Oligo 4 extended to 1 μs, by comparing the beginning -from 50 to 150ns- and the end -from 900 to 1000ns- of trajectories (S3 Fig). This analysis produced very similar BII percentages on each step of Oligo 4, with only slight differences (8% for the worst case) on some BII-rich steps. Thus, the first 100ns of production (from 50 to 150ns), while not sufficient to ensure a complete convergence, surprisingly offer a rather good estimation of the backbone behavior, supporting the expectation that the simulations are essentially converged for practical purposes with respect to the BI/BII balance on the 450ns time scale.

We recall that the MDs were performed restraining the Watson-Crick hydrogen bonds in the first and last base pairs. We chose this protocol since, with unrestrained MDs, convergence issues were observed in conjunction with fraying events involving larger than expected motions of the terminal regions, consonant with previous reports [5, 13, 48]. Since the structural signature of these long-lived fraying events in the unrestrained MDs is not supported by the NMR measurements (see “Restrained base pairing on the first and last base pairs” in Materials and Methods), Watson-Crick pairing restraints of the first and last base pairs were applied in the analyzed MDs. This remedied the convergence concerns otherwise observed in the unrestrained MDs (see the example of restrained and unrestrained 1 μs P-MDs of Oligo 4 in S4 Fig). Importantly, however, the BII percentages calculated on the central part (N_3_→N_10_•N_15_→N_22_) of the dodecamers are quasi identical (correlation coefficient of 0.98) in both restrained and unrestrained MDs. This indicates that the behavior of the internal steps was reproducible in different conditions, another reassuring element concerning the convergence of the simulations presented here.

Overall, we observe that the DNA backbone dynamics is essentially converged with MDs of several hundredth ns. This convergence timeframe is realistic considering that the phosphate groups undergo rapid (nano-picosecond timescale) conformational exchange according to NMR [49, 50]. Convergence in terms of sampling of the backbone states allows us to concentrate the following analysis on the influence of the force-fields.

### Definition of the BI and BII states for MD analyses

In free DNA X-ray structures the distribution of the pseudo-angle (ε-ζ) is characterized by two major peaks centered around (ε-ζ) = -90° (BI, ε in *trans* and ζ in *g-*) and (ε-ζ) = 90°, (BII, ε in *g-* and ζ in *trans*) (S5 Fig). Between these two maxima, a region covering (ε-ζ) values from -60 to +70° contains phosphate linkages with ε:*trans* typical of BI and ζ:*trans* typical of BII. Therefore this region may be considered ambiguous in terms of BI/BII categorization. The separation between BI and BII is commonly set at the minimum of the (ε-ζ) distribution, which is close to (ε-ζ) = 0 in the X-ray distribution (S5 Fig). BI and BII are thus usually characterized by negative and positive (ε-ζ) values, respectively.

**Fig 2:**
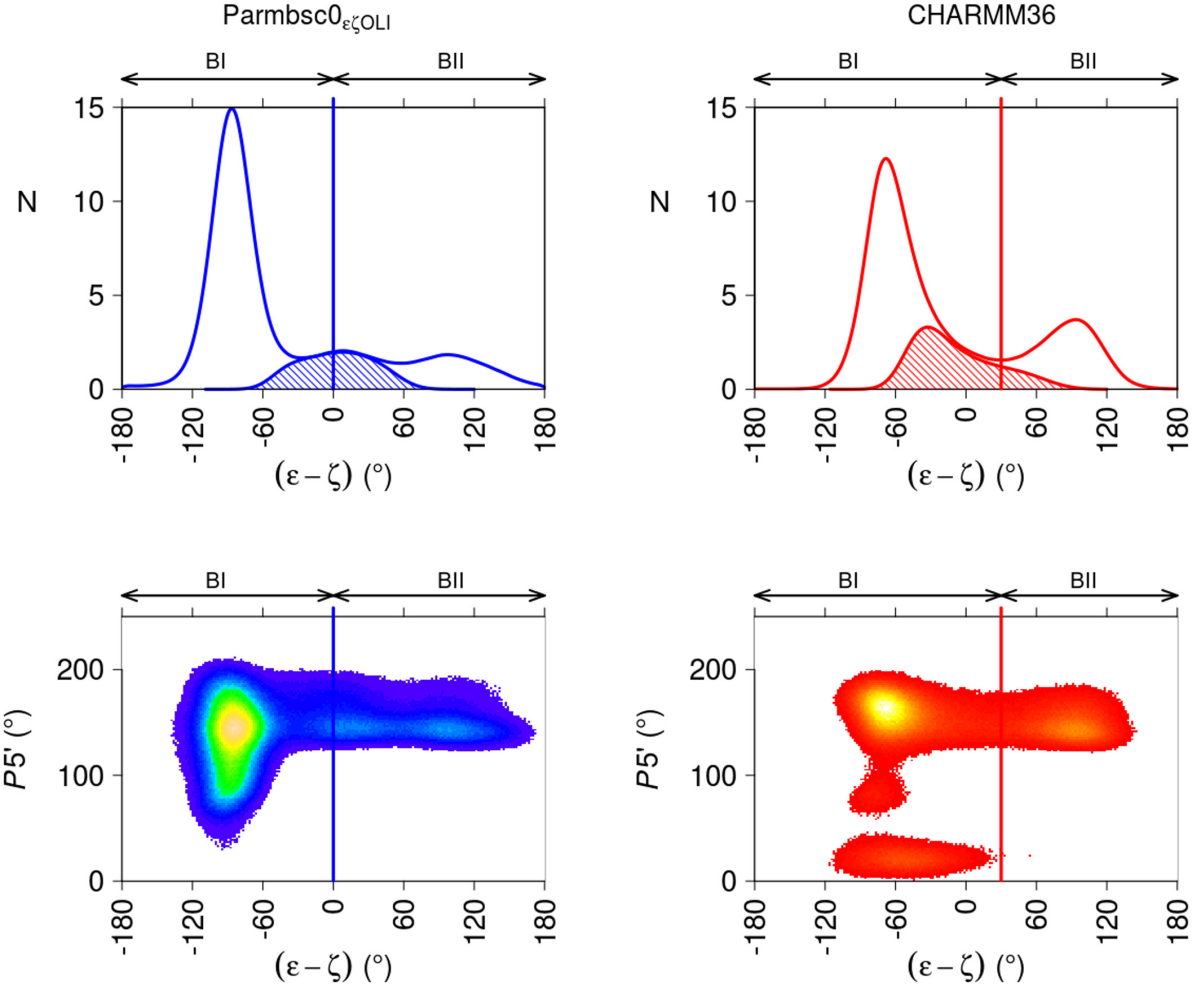
Distribution of backbone (ε-ζ) values and 5’ sugar behavior in MD snapshots. Top panels: the frequencies (N) of (ε-ζ) values were extracted from P-MDs (left panel, blue line) and C-MDs (right panels, red line) snapshots. The BI and BII regions are indicated, in accordance with the analysis presented in the main text. The hashed regions correspond to the distribution of ε/ζ:trans/trans, the categorization of which is ambiguous with regard to BI or BII. Bottom panels: pseudorotation phase angle (P) of the 5’ sugar puckers as a function of (ε-ζ) values (°), extracted from P-MDs (left panel) and C-MDs (right panel). The sugar ring conformations were in *north* (pseudorotation phase angle 0 to 50°), *east* (50 to 120°) or *south* (120 to 220°). The (ε-ζ) region assigned to BII does not contain 5’ *north* sugars. The color gradient is indicative of the density, with the highest densities in yellow. The vertical lines indicate the (ε-ζ) values used here to separate BI from BII in P- (blue lines) and C-MDs (red lines).

The pattern observed in the X-ray structures for the (ε-ζ) distribution is globally preserved in P-MDs and C-MDs, while influenced by the force-fields (Fig 2). Thus, the operational definition of the BI and BII states in MDs must be carefully scrutinized, and possibly adapted. In addition to the (ε-ζ) histograms, the sugar populations in the *south*, *east* and *north* puckers (e.g. Oligo 4 sugars in S6 Fig) were considered, since this criterion is relevant to the definition of BI and BII. Indeed, crystallographic and NMR investigations established that BI is tolerant in terms of surrounding 5’ and 3’ sugar puckers, while BII is restricted to *south* puckers, especially with respect to 5' sugars [15, 18, 50].

The (ε-ζ) histogram of C-MDs has a minimum at (ε-ζ) = 30°, located at a tail of the ε/ζ:*trans/trans* region (Fig 2). 5’ *south* sugars are observable in both BI and BII regions; 5' *east* sugars fall in (ε-ζ) from -110 to -50°, inside the conventional BI region; 5' *north* sugars are associated to a larger range of (ε-ζ) values, but are suppressed above (ε-ζ) = 30° (Fig 2). This sugar behavior and the minimum of (ε-ζ) at 30° offer an analogy with the X-ray observations, such that (ε-ζ) = 30° was deemed suitable to separate BI from BII with CHARMM36. Using instead the conventional cutoff (ε-ζ = 0) to separate BI from BII would not result in a dramatically different description, since the average BII population inferred with (ε-ζ) > 0° would only be 4% higher than that based on (ε-ζ) > 30°. However, in view of the distribution of 5’ *north* sugars, the criterion (ε-ζ) > 30° was preferred to define BII with CHARMM36. Incidentally, the present C-MD data confirm the strong association between BII and 5’ *south* sugars.

With Parmbsc0_εζOLI_, no clear minimum is observed in the (ε-ζ) distribution between the BI and BII peaks, which are separated by a flat (ε-ζ) distribution (Fig 2). This intermediate region, centered around (ε-ζ) = 0, contains ε/ζ:*trans/trans* conformers (Fig 2), which represent 10% of the snapshots. In absence of a clear minimum in the (ε-ζ) distribution, histograms of ε and ζ were considered. This approach was adopted in previous Parmbsc0 trajectories [15, 18, 50, 51], where the BII linkages were defined relative to the minimum of the distribution. Here, with the minimum of the ζ distribution at ζ = 230°, this approach would designate as BII the range above (ε-ζ) = -50°, a strongly negative value. Conversely, the transition from BI to BII would be at (ε-ζ) = 40° if chosen to be at the minimum of the ε histogram (ε = 240°). So, the ε and ζ histograms do not provide a coherent definition of BI and BII ranges for P-MDs here. In addition, the sugar dynamical regime is of little help since Parmbsc0_εζOLI_ generates only a few *north* sugars (Fig 2 and S6 Fig). In absence of any convincing more specific rationale to assign the ε/ζ:*trans/trans* snapshots to either BI or BII with Parmbsc0_εζOLI_, we adopted the common BII definition, (ε-ζ) > 0°. Such a decision is somewhat arbitrary but the uncertainty it introduces is limited. Indeed, shifting the (ε-ζ) dividing value by 20° ((ε-ζ) = -20° or +20°) only changed the BII population by +/-2%. In sum, BII percentages were extracted using (ε-ζ) > 0° for P-MDs and (ε-ζ) > +30° for C-MDs throughout this work.

These considerations illustrate the difficulty in defining BI and BII unambiguously, in a manner which would be meaningful and transferable across different structural models. It also draws the attention to some differences between Parmbsc0_εζOLI_ and CHARMM36 regarding their representations of sugars and the (ε-ζ) distribution. Yet, the following sections show that a consistent overall picture emerges from CHARMM36 and Parmbsc0_εζOLI_.

### Overall BII populations from MD simulations compared to NMR

The four dodecamers studied here correspond to 72 dinucleotide steps, excluding the terminal steps. The corresponding 72 ^31^P chemical shifts (δPs) were measured and converted to BII percentages, BII%_*from NMR*_, using an empirical procedure based on a calibration involving a comparison of NMR and X-ray structural data [24] (see also Materials and Methods). In this procedure, δPs of pure BI and pure BII states are assumed to be sequence-independent, even if they could be modulated by the dinucleotide sequence, as suggested by a computational study [52]. However, previous studies showed that neglecting subtle sequence effect on δPs of pure BI and pure BII produced reasonable estimations [27, 53], for instance with points where BI and BII are expected to be equally populated [27]. Another indication of the protocol reliability is the consistency between the average BII percentage either derived from the average δPs of the 72 steps considered here (19% of BII, from δP_av_ = -4.20 ppm at 30°) or inferred from statistics of X-ray structures (20% of BII) [15].

A first test of the force fields is to compare the NMR-inferred and simulated BII populations, averaged on the 72 dinucleotides. The simulated overall average BII percentages are 11% in P-MDS and 18% in C-MDs. Thus, Parmbsc0_εζOLI_ somewhat underestimated the BII populations, as noted before [13]. The excellent agreement of the CHARMM36 value with experiment is an obvious improvement compared to CHARMM27, which severely underrepresented BII [9, 11].

That the force fields, in particular CHARMM36, produce overall BII population commensurate with experimental data is very encouraging, considering that the treatment of the backbone by previous force-fields fell outside the experimental range. Since the dinucleotides have markedly different propensities to populate BII [15, 27, 28, 51, 53], reproducing the sequence effects is a more stringent test of the force-fields, examined in the following.

### Sequence-dependent BII propensities from simulations versus NMR

A previous dataset of 323 measured δPs has established that the 16 dinucleotides (NpN steps for a single strand in a duplex context) composing B-DNA are associated with specific δP values [28]. Since δP translates into a BII propensity it implies that the BI/BII populations are primarily controlled by the dinucleotide sequence [28]. The additional 72 δPs considered here conform to this sequence pattern, validating the notion of dinucleotide-specific BII propensity [27]. Thus, the sequence-dependent BII populations derived from δPs provide a rare opportunity to test the sequence-dependent behavior of DNA force-fields in solution. One notes that adjustments made to Parmbsc0_εζOLI_ and CHARMM36 to increase the BII populations were not tailored depending on the base sequence, in contrast with, for instance, the CMAP approach [54]. In other words, the same backbone force-field parameters are applied to any sequence. Therefore, differences in the backbone behavior during simulations can only be ascribed to intrinsic sequence-dependent properties.

**Fig 3:**
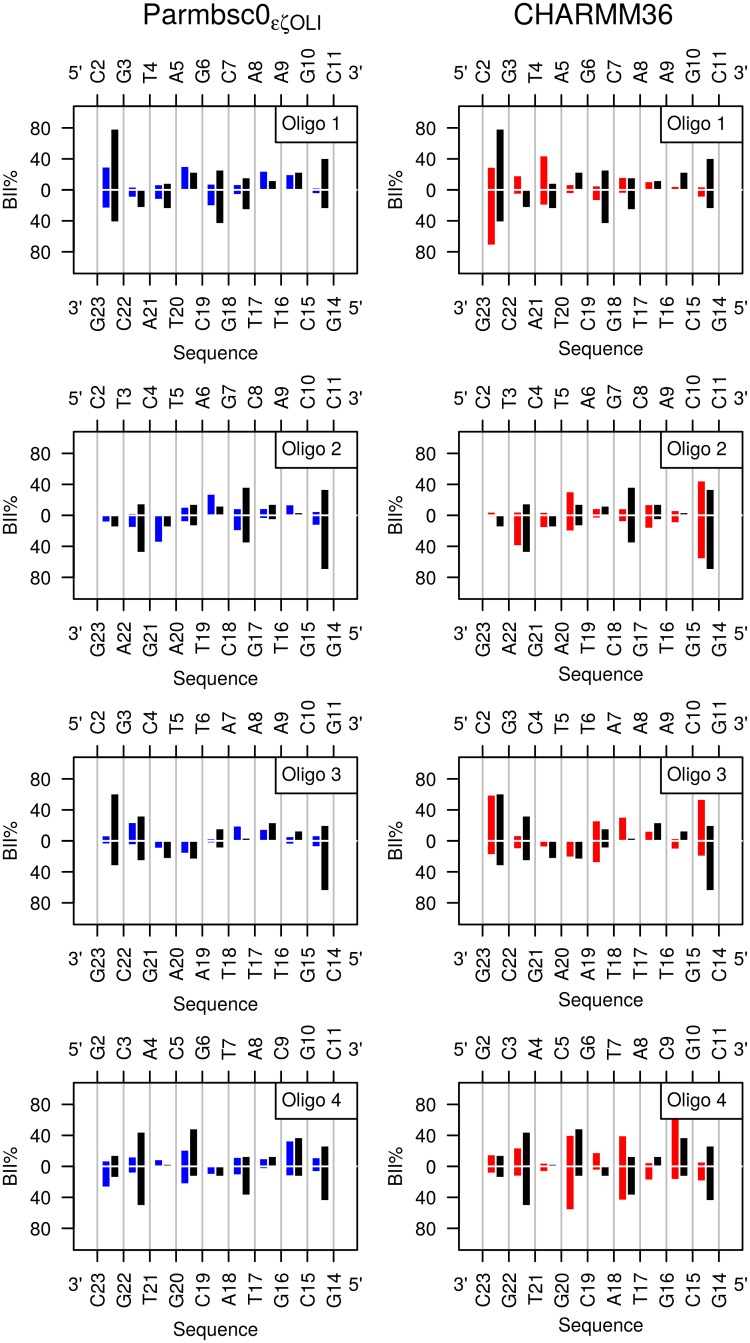
Comparison between simulated and experimental BII percentages along the four dodecamers. BII percentages (BII%) were plotted along the two complementary strands of each dodecamer sequence. BII% were extracted from P-MDs (left panels, blue) and C-MDs (right panels, red), or inferred from the 72 δPs collected by NMR (black). The error on BII% from NMR was estimated to be ±10%.

To examine whether Parmbsc0_εζOLI_ and CHARMM36 reproduce the effect of sequence on BII populations, the simulated BII percentages were compared to their experimental counterparts, considering the individual phosphates (BII%_*from MD*_ versus BII%_*from NMR*_, given in S1 Table). BII%_*from MD*_ and BII%_*from NMR*_ are overall moderately correlated (Table 2 and S7 Fig). The simulated BII percentages of half of the 72 steps (53% for both P-MDs and C-MDs) are within BII%_*from NMR*_ ±10%, where the 10% interval corresponds to the tolerance allowed around the NMR-based BII percentages (see Materials and Methods). The comparison between BII%_*from NMR*_ and BII%_from MD_ is shown in Fig 3 for each non-terminal phosphate of the four dodecamers.

**Table 2:** BII percentages from NMR compared to their simulated counterparts.

	P-MDs		C-MDs	
	CC	Δ_av_	CC	Δ_av_
all	0.42	14 (14)	0.47	14 (12)
Oligo 1	0.62	14 (13)	0.33	18 (14)
Oligo 2	0.31	14 (15)	0.75	10 (8)
Oligo 3	0.32	14 (17)	0.49	14 (12)
Oligo 4	0.36	13 (14)	0.35	16 (13)

The linear correlation coefficient (CC) were calculated between BII percentages (BII%) inferred from experimental δPs and those extracted from the simulations with Parmbsc0_εζOLI_ (P-MDs) and CHARMM36 (C-MDs). Δ_av_ represents the average difference between the simulated and experimental BII% values; standard deviations are in brackets.

A more detailed analysis, in particular on the dinucleotides present in several occurrences in the dodecamers, shows that Parmbsc0_εζOLI_ and CHARMM36 correctly reproduce the low or moderate BII%_*from NMR*_ (< 20%) of CpT, GpT, ApC, ApG, ApA and TpT (Fig 3 and Table 3 for the steps present in several occurrences in the dodecamers). However, this overall agreement suffers some shortcomings. The BII population of TpA tends to be either underestimated or overestimated with Parmbsc0_εζOLI_ and CHARMM36, respectively. GpC steps in BII are quasi systematically underestimated by both force fields, as well as one of the three CpA•TpG in Oligo 4. Parmbsc0_εζOLI_ generated too low BII percentages on CpC and GpG in Oligo 2 and most CpG (seven on a total of ten). In C-MDs, the representation of CpC, GpG and CpG is reasonable whereas inversions of BII% occurs at two complementary CpG•CpG steps. Indeed, the NMR gives asymmetrical BII percentages for C_2_pG_3_•C_22_pG_23_ in Oligo 1, with 79% of BII for C_2_pG_3_ and 42% for C_22_pG_23_. C-MD gave the reverse, with 30% and 75% of BII for C_2_pG_3_ and C_22_pG_23_, respectively. A similar situation arises for C_10_pG_11_•C_14_pG_15_ in Oligo 3.

**Table 3:** BII percentages from NMR compared to their simulated counterparts for selected steps.

	N	BII%_*from NMR*_	BII%_*from P-MDs*_	BII%_*from C-MDs*_
CpT	6	0 (0)	0 (0)	4 (2)
GpT	6	0 (0)	3 (1.5)	14 (5.5)
TpT	4	0 (0)	0 (0)	2 (1)
ApC	6	12 (7)	10 (3)	5 (1)
ApA	4	16 (10)	19 (4)	18 (9)
TpA	8	17 (9.5)	9 (4)	32 (10)
ApG	6	19 (5)	22 (11)	8 (5)
GpC	12	31 (10)	12.5 (8)	10 (4)
CpG	10	41 (22)	17 (10.5)	43 (21)

The BII percentages inferred from experimental δPs or extracted from the simulations with Parmbsc0_εζOLI_ (P-MDs) and CHARMM36 (C-MDs) are reported for the steps present in more than 3 occurrences (N) in the dodecamers. The values are averaged, with standard deviations in brackets.

Our results confirm that adjustments specifically aimed at enhancing access to the BII state produce convincing, positive effects, especially perceptible in CHARMM36. That the increase in simulated BII populations is not distributed uniformly along the sequences (Fig 3) is not trivial since, as noted above, the computational models were not parametrized to reproduce the BII% for specific dinucleotides, but were only adjusted to be generically more permissive to BII. Admittedly, discrepancies still exist between the experimental sequence effect on the BI↔BII equilibrium and Parmbsc0_εζOLI_ or CHARMM36. However, an essential point is that the simulations are now sufficiently BII-rich to extend the analysis to aspects of the backbone dynamics that eludes experimental approaches.

### New insights from the simulations: Independence of the states of facing phosphate groups

The phosphate groups facing each other across the strands can adopt homogeneous combinations, BI•BI or BII•BII, or hybrid combinations, BI•BII or BII•BI (denoted here BI•BII|BII•BI, where the vertical bar means logical “or”). The populations of these combinations are especially meaningful from the point of view of B-DNA mechanics, because inter base pair parameter values are associated to the conformational states of two facing phosphate linkages [15, 16, 24, 40, 55–58], as also addressed below.

**Fig 4:**
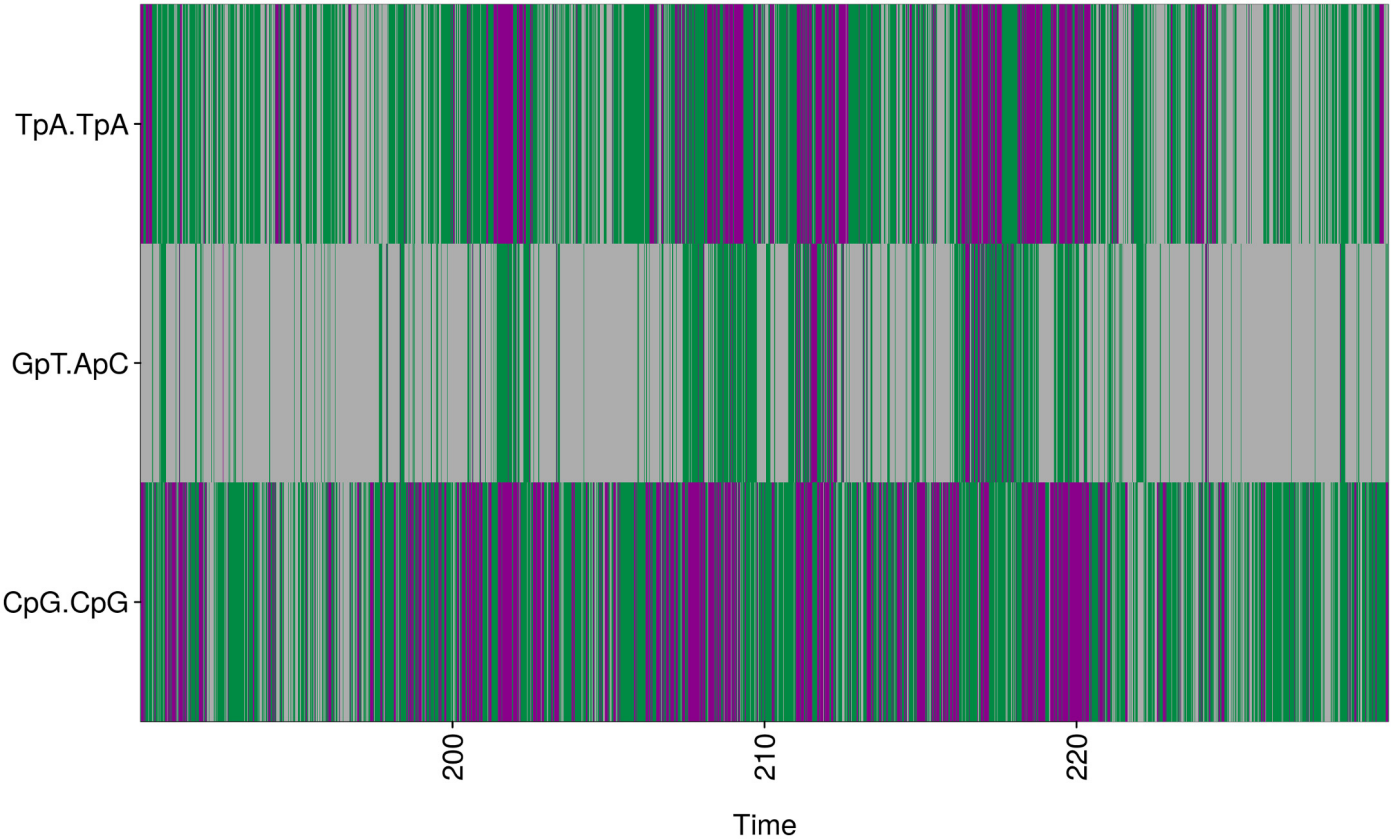
Conformational combinations of facing phosphate linkages illustrated with CHARMM36. In C-MDs, the BII-rich facing phosphate linkages adopt three conformational combinations, BI•BI (grey), BI•BII|BII•BI (green) and BII•BII (violet). The course of these combinations versus time (ns) is illustrated for facing phosphate groups of Oligo 4, two BII-rich steps, C_5_pG_6_•C_19_pG_20_ and T_7_pA_8_•T_17_pA_18_, and one BI-rich step, G_6_pT_7_•A_18_pC_19_. For clarity, only a small part of the trajectory is shown here.

The behavior of facing phosphate linkages cannot be deduced from δP measurements, which report time and ensemble-averaged BII percentages for individual phosphates. The present simulations offer the opportunity to inspect the dynamic behavior of phosphate linkages in complementary dinucleotides and to estimate possible correlation. Indeed, several steps in C-MDs, in particular CpG•CpG, CpC•GpG and TpA•TpA, adopt the three combinations, BI•BI, BI•BII|BII•BI or BII•BII (Table 4 and Fig 4). In P-MDs, BI•BI and BI•BII|BII•BI are also frequently observed, but the BII•BII populations are almost inexistent (Table 4), consistent with Parmbsc0_εζOLI_ generating fewer BII conformers than CHARMM36.

**Table 4:** Conformational combinations of facing phosphates in MDs.

		(BI•BI)%		(BI•BII|BII•BI)%		(BII•BII)%	
	N	P-MDs	C-MDs	P-MDs	C-MDs	P-MDs	C-MDs
CpG•CpG	5	67 (18)	27 (7)	31 (17)	58 (5)	1 (1)	14 (6)
CpC•GpG	1	82	26	18	47	1	27
TpA•TpA	4	83 (7)	46 (11)	17 (7)	44 (7)	0	10 (4)
GpC•GpC	6	76 (9)	81 (4)	23 (9)	18 (4)	1 (1)	1 (0)

The percentages of the conformational combinations of facing phosphate linkages, BI•BI, BI•BII|BII•BI, and BII•BII, are reported for selected steps with substantial BII% during either P-MDs or C-MDs (see Fig 3). The values corresponding to CpG•CpG, TpA•TpA, and GpC•GpC steps, present in several occurrences (N) in the dodecamers, are averaged, with standard deviations in brackets.

**Fig 5:**
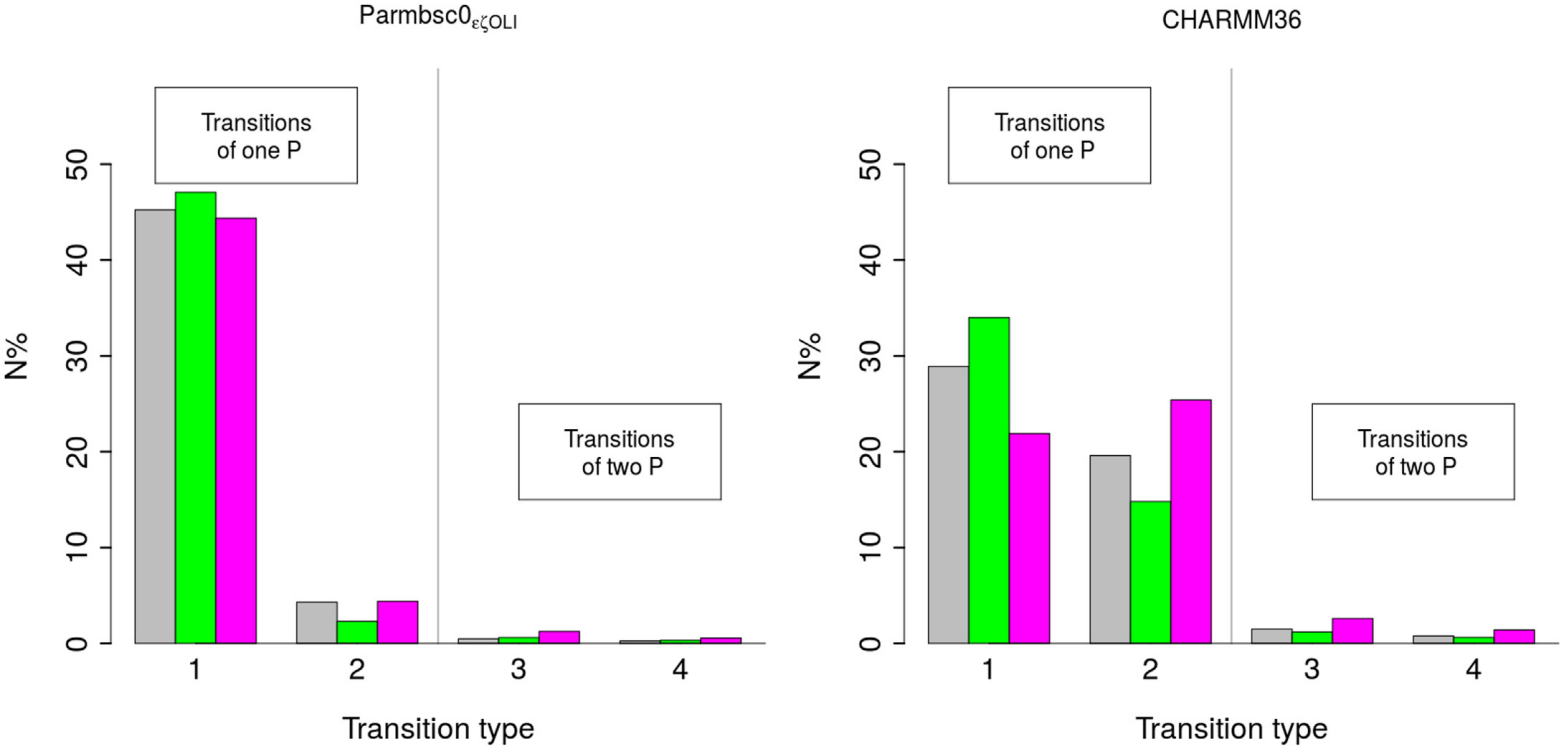
Statistics on the transitions between the conformational states of facing phosphate groups. The transitions between facing phosphate group combinations were analyzed for C_5_pG_6_•C_19_pG_20_ (grey) and T_7_pA_8_•T_17_pA_18_ (green) in Oligo 4, and C_10_pC_11_•G_14_pG_15_ (pink) in Oligo 2, from P-MDs (left) and C-MDs (right). These steps were chosen because they adopt BII•BII in the simulations. N% is the percentage of a transition type relative to the total number of transitions. The transition types are labeled as follow: 1: BI•BI → BI•BII|BII•BI or the inverse, BI•BII|BII•BI → BI•BI; 2: BI•BII|BII•BI → BII•BII or BII•BII → BI•BII|BII•BI; 3: BI•BII → BII•BI or BII•BI → BI•BII; 4: BI•BI → BII•BII or BII•BII → BI•BI.

Fig 5 illustrates the statistics of the transitions between the facing phosphate combinations for steps that adopt BII•BII in both P-MDs and C-MDs. The same result holds for any other complementary step investigated here in which the facing phosphates undergo BI ↔ BII transitions. With both force fields, the large majority of the transitions between BI•BI, BI•BII|BII•BI and BII•BII involves only one of the two facing phosphates (BI•BI ↔ BI•BII or BII•BI; BI•BII or BII•BI ↔ BII•BII). BI•BII|BII•BI ↔ BII•BII are infrequent in P-MDs, the BII•BII state being poorly populated. In both P-MDs and C-MDs, the simultaneous transitions of two phosphate states (BI•BII ↔ BII•BI; BI•BI ↔ BII•BII) are very rare, representing at most 5% of the total number of transitions (Fig 5).

The populations of BI•BI, BI•BII|BII•BI and BII•BII can be addressed with simple elements from probability theory, summarized here before comparison to the MD data. *P*_*i*_(BII) is the probability that phosphate *i* is in BII, the complementary event has probability *P*_*i*_(BI) = 1 –*P*_*i*_(BII). The states of facing phosphate pairs *i*,*j* are characterized by pair probability distributions, *P*_*i*,*j*_(BI•BI), *P*_*i*,*j*_(BI•BII), *P*_*i*,*j*_(BII•BI) and *P*_*i*,*j*_(BII•BII). Because the facing phosphate groups are either BI or BII, the probabilities satisfy the relations:

$$P_{i,j}\left(\text{BI}•\text{BI}\right)\;+\;P_{i,j}\left(\text{BI}•\text{BII}\right)\;=\;P_{i}\left(\text{BI}\right)$$

$$P_{i,j}\left(\text{BI}•\text{BI}\right)\;+\;P_{i,j}\left(\text{BII}•\text{BI}\right)\;=\;P_{j}\left(\text{BI}\right)$$

$$P_{i,j}\left(\text{BII}•\text{BI}\right)\;+\;P_{i,j}\left(\text{BII}•\text{BII}\right)\;=\;P_{i}\left(\text{BII}\right)$$

$$P_{i,j}\left(\text{BI}•\text{BII}\right)\;+\;P_{i,j}\left(\text{BII}•\text{BII}\right)\;=\;P_{j}\left(\text{BII}\right)$$

Summing the last two equations gives:

$$\left[P_{i,j}\left(\text{BII}•\text{BI}\right)\;+\;P_{i,j}\left(\text{BI}•\text{BII}\right)\right]\;+\;2\;P_{i,j}\left(\text{BII}•\text{BII}\right)\;=\;\left[P_{i}\left(\text{BII}\right)\;+\;P_{j}\left(\text{BII}\right)\right]$$ (1)

The first term on the left in (1) is the probability of BI•BII|BII•BI, *P*_*i*,*j*_(BII•BI|BI•BII). Note that here *P*_*i*,*j*_(BII•BI|BI•BII) does not denote a conditional probability, but simply the probability of states BII•BI or BI•BII. So Eq 1 is equivalent to

$$P_{i,j}\left(\text{BII}•\text{BI}|\text{BI}•\text{BII}\right)\;+\;2\;P_{i,j}\left(\text{BII}•\text{BII}\right)\;=\;P_{i}\left(\text{BII}\right)\;+\;P_{j}\left(\text{BII}\right).$$ (2)

Eq 2 is general, as it follows directly from the definitions and it does not rely on any assumption about the independence (correlation) of facing phosphate groups.

One now examines the case when the states of the two facing phosphate groups are independent of each other. Then, the pair probabilities factorize: *P*_i,j_(*b*•*b'*) = *P*_i_(*b*) *P*_j_(*b'*), where *b* and *b’* stand for any of the phosphate states. In particular, we have

$$P_{i,j}\left(\text{BII}•\text{BII}\right)\;=\;P_{i}\left(\text{BII}\right)P_{j}\left(\text{BII}\right)$$ (3)

$$P_{i,j}\left(\text{BII}•\text{BI}|\text{BI}•\text{BII}\right)\;=\;P_{i}\left(\text{BII}\right)\;+\;P_{j}\left(\text{BII}\right)\;-\;2P_{i}\left(\text{BII}\right)P_{j}\left(\text{BII}\right).$$ (4)

Eq 4 follows from Eqs (2) and (3) and from the relations: *P*_*i*_(BI) = 1- *P*_*i*_(BII) and *P*_*j*_(BI) = 1- *P*_*j*_(BII).

Eqs 3 and 4 mean that, under the assumption of statistical independence of the two individual facing phosphates, the knowledge of the single phosphate probabilities *P*_*i*_(BII) and *P*_*j*_(BII) is sufficient to find the probabilities of BII•BI|BI•BII, BII•BII, and then also of BI•BI by using equation:

$$P_{i,j}\left(\text{BI}•\text{BI}\right)\;=\;1–\;[P_{i,j}\left(\text{BII}•\text{BI}|\text{BI}•\text{BII}\right)\;+\;P_{i,j}\left(\text{BII}•\text{BII}\right)].$$

**Fig 6:**
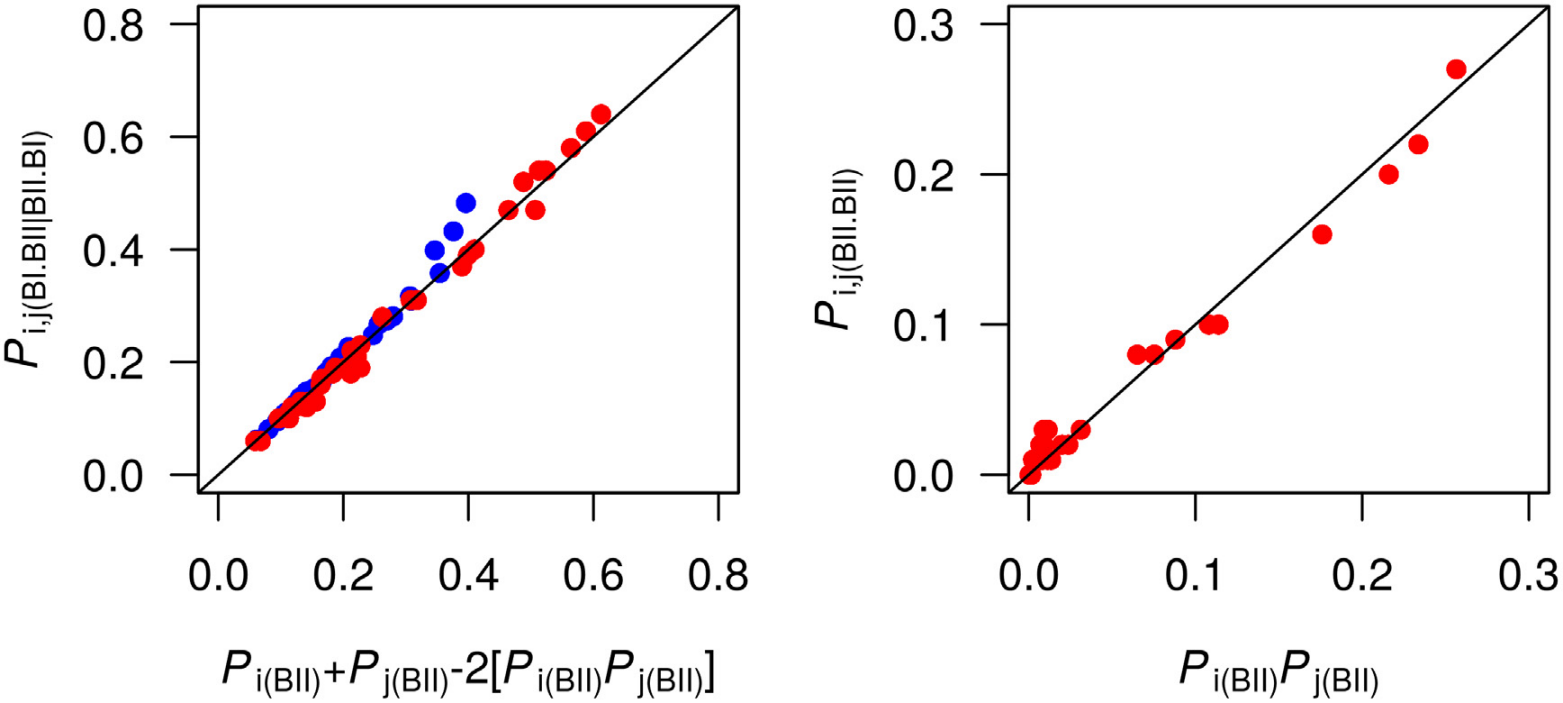
Facing phosphate states pair correlations. The facing phosphate states BI•BII|BII•BI and BII•BII have probabilities *P*_*i*,*j*_(BII•BI|BI•BII) (left panel) and *P*_*i*,*j*_(BII•BII), (right panel), respectively, extracted from P-MDs (blue) and C-MDs (red) for each complementary step in the dodecamers. *P*_*i*,*j*_(BII•BII) versus *P*_*i*_(BII) P_j_(BII) is not reported for P-MDs because of the much reduced BII•BII populations in these simulations. These *P*_*i*,*j*_ probabilities are compared to expressions containing the individual BII probabilities, *P*_*i*_(BII) and *P*_*j*_(BII), under the assumption that the conformational states of facing phosphate groups are independent of each other Eq 4 for *P*_*i*,*j*_(BII•BI|BI•BII) and Eq 3 for *P*_*i*,*j*_(BII•BII), see main text). The diagonal lines correspond to y = x.

The next step was to test the possibility of uncorrelated facing phosphates against data collected from the MDs. Thus, *P*_*i*,*j*_(BII•BI|BI•BII), *P*_*i*,*j*_(BII•BII), *P*_*i*_(BII) and *P*_*j*_(BII) were evaluated as the proportions of these states in the MD trajectories; *P*_*i*,*j*_(BII•BI|BI•BII) was compared to [*P*_*i*_(BII) + *P*_*j*_(BII)−2*P*_*i*_(BII) *P*_*j*_(BII)] in P-MDs and C-MDs; *P*_*i*,*j*_(BII•BII) was compared to [*P*_*i*_(BII) *P*_*j*_(BII)] in C-MDs only, since they generate sizable BII•BII populations in contrast with P-MDs. The agreement between the compared quantities is clearly visible in Fig 6, with correlation coefficients of 0.99. That is, the distribution of BII steps between the BI•BII|BII•BI and BII•BII combinations in complementary dinucleotide matches Eqs 3 and 4 very well. Thus, the conformational states of the facing phosphates are statistically independent of each other.

In sum, the ability of both Parmbsc0_εζOLI_ and CHARMM36 to generate phosphates visiting BII enabled to gain new insights into their dynamics and populations. Thus, simultaneous transitions of two facing phosphate groups are very rare. The two force-fields unambiguously support the notion of statistical independence of the conformational states of individual, facing phosphates. This means that the populations of the three combinations of facing phosphates can be simply expressed from the BII propensities of individual phosphate groups, in particular from experimental data, as developed in the next section.

### Quantifying the facing phosphate combinations from NMR

**Fig 7:**
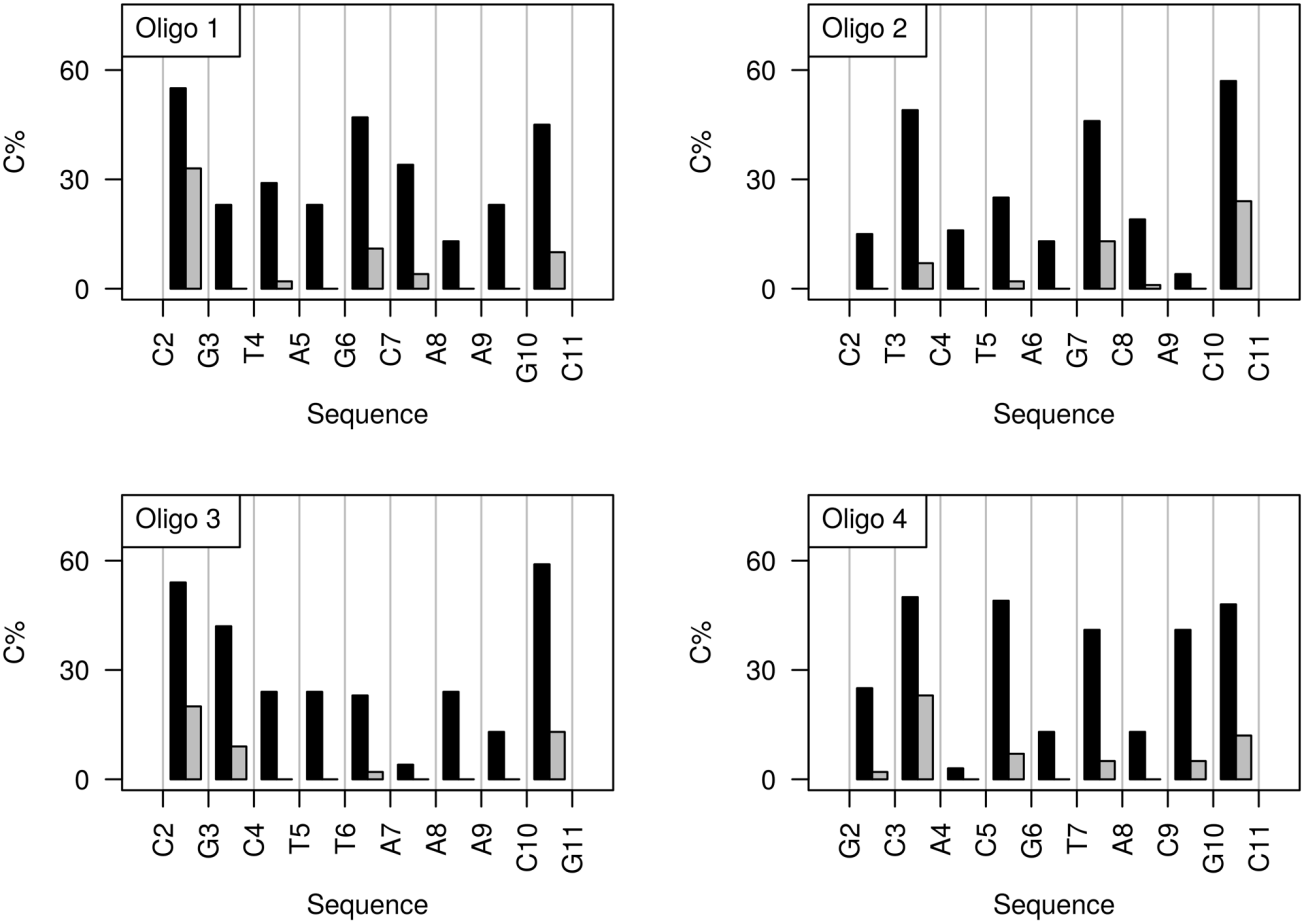
Quantification of the conformational combinations of independent facing phosphates based on NMR data. The percentages of BI•BII|BII•BI (black bars) and BII•BII (grey bars) combinations (C%) of facing phosphates are plotted along the four dodecamer sequences. These percentages were calculated in the regime of independence of the conformational states of facing phosphates with Eqs 3 and 4 and the NMR data. The values are given in S1 Table.

Considering that the notion of uncorrelated facing phosphate is convincing, the probabilities of states BI•BII|BII•BI and BII•BII were calculated using Eqs 3 and 4, respectively, and equating *P*_*i*_(BII) and *P*_*j*_(BII) to *P*_*i from NMR*_ (BII) and *P*_*j from NMR*_ (BII) (equivalent to BII%_*from NMR*_ given in S1 Table). This has the advantage to use the experimental data directly (δP-derived BII percentages) to quantify the phosphate states, bypassing the limitations in the simulated estimates of the phosphate state populations. The resulting experimentally inferred BI•BII|BII•BI and BII•BII populations along the four dodecamers are shown in Fig 7 and the values are given in S2 Table. According to this approach, most CpG•CpG and GpC•GpC, as well as the only CpC•GpG, are characterized by high percentages (45% and more) of BI•BII|BII•BI (Fig 7). CpG•CpG in Oligos 1 and 3, CpC•GpG in Oligo2 and CpA•TpG in Oligo 4 are in addition more than 20% in BII•BII (Fig 7). Overall, BI•BI is not the most frequent state in 12 steps, out of a total of 36, in the four dodecamers.

As seen above (Fig 3), the individual BII percentages extracted from MDs differ from those inferred from NMR; accordingly, the corresponding respective populations of BI•BII|BII•BI and BII•BII are not identical. However, the match between C-MD and experimentally inferred data is reasonable (S8 Fig), with correlation coefficients of 0.62 for BI•BII|BII•BI and 0.57 for BII•BII. So, CHARMM36 appears to represent the sequence-dependent behavior of the pairs of facing phosphates better than that of individual phosphates (Table 2). This improvement reflects in part compensatory effects between the two strands of CpG•CpG steps, in which the asymmetric individual BII percentages are inversed in C-MDs and NMR (see the above section “Sequence-dependent BII propensities from simulations versus NMR”).

Overall, the realization that the states of facing phosphates are independent enables to derive their populations from δP-based BII percentages. Applying this approach reveals that all the complementary dinucleotides in the four dodecamers populate both BI•BI and BI•BII|BII•BI, some of them also display significant percentages of BII•BII (Fig 7 and S2 Table). This prevalence of BII-containing steps is of real importance for the DNA intrinsic mechanics, as examined next.

### Conformational combinations of facing phosphate and inter base pair parameters

BI•BI, BI•BII|BII•BI or BII•BII are associated to different values of slide, roll and twist in X-ray structures [15, 16, 39]. However, the requirement to select only very high resolution X-ray structures to ensure the accuracy of backbone dihedral angles [59] drastically limits the data for analysis. A previous study [28] underlined that BII conformers in such X-ray structures occur almost exclusively in CpG, CpA, TpG, GpG, and GpC; furthermore, the BII•BII combination was only observed in CpA•TpG. The improved representation of the DNA backbone with Parmbsc0_εζOLI_ and CHARMM36 offers the opportunity to broaden the analysis of the helicoidal parameters associated to the facing phosphate combinations for a larger variety of complementary dinucleotides than in X-ray datasets. Consistent results between both force fields would of course strengthen the conclusions.

The mean values of the six inter base pair parameters (shift, slide, rise, slide, roll and twist) were calculated for each conformational combination of the facing phosphate groups, after merging all equivalent conformational combinations across complementary steps. Slide, roll and twist are found very sensitive to the facing backbone conformational combinations (Table 5) contrary to invariant shift and tilt (S3 Table). As in a previous study using Parmbsc0 [40], rise variations are observed, but the change between BI•BI and BII•BII does not exceed 0.2 Å in both P-MDs and C-MDs (S3 Table).

**Table 5:** Inter base pair parameters variations associated to the conformational states of the facing phosphate groups.

	Slide (Å)		Roll (°)		Twist (°)	
	P-MDs	C-MDs	P-MDs	C-MDs	P-MDs	C-MDs
BI•BI	-0.2 (0.3)	-0.2 (0.2)	4 (3.7)	3.8 (3.7)	31.9 (1.9)	33.7 (3.0)
BI•BII|BII•BI	0.2 (0.3)	0.2 (0.2)	-0.5 (3.3)	-3.0 (3.6)	37.9 (2.3)	36.5 (2.6)
BII•BII	0.6 (0.3)	0.6 (0.2)	-7 (4.3)	-9.9 (4.1)	41.7 (3.8)	39.3 (2.6)
Δ(BII•BII−BI•BI)	+0.8	+0.8	-11	-13.7	+9.8	+5.6

Values of Slide, Roll and Twist generated by Parmbsc0_ε_ζ_OLI_ (P-MDs) and CHARMM36 (C-MDs) for the three conformational combinations of facing phosphate groups, extracted after merging data from all simulated complementary dinucleotides in the four dodecamers. Standard deviations are in brackets. The last row reports the difference between the inter base pair parameters in the two extreme situations, BI•BI and BII•BII.

**Fig 8:**
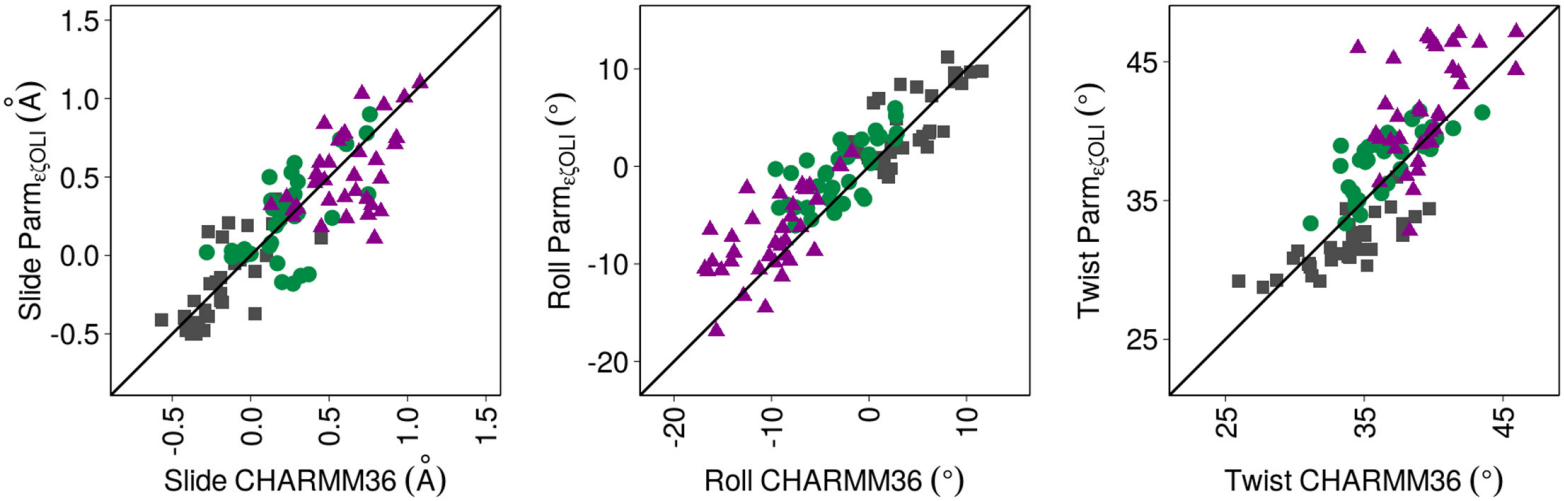
Slide, Roll and Twist values generated by Parmbsc0_εζOLI_ and CHARMM36 for individual complementary dinucleotide steps, versus the facing phosphate combinations. The mean values of Slide, Roll and Twist were calculated over the MDs for each of the 36 complementary dinucleotides of the four dodecamers, categorized according to the BI•BI (grey squares), BI•BII|BII•BI (green circles) and BII•BII (violet triangles) combinations of their facing phosphate groups. The data were extracted from P-MDs and C-MDs, and time-averaged for each conformational combination. The standard deviations are 0.6 for slide, ~7° for roll and 6° for twist, with both force-fields. The correlation coefficients are 0.84 (P-slide versus C-slide), 0.90 (P-roll versus C-roll) and 0.78 (P-twist versus C-twist). The diagonal lines correspond to y = x.

It is striking that there is almost quantitative agreement on the changes of slide, roll and twist across BI•BI, BI•BII|BII•BI and BII•BII with both force-fields (Table 5). Yet, CHARMM36 does not increase the twist as much as Parmbsc0_εζOLI_ from BI•BI to BII•BII. Considering the 36 individual complementary dinucleotides of the four oligomers (Fig 8) confirms this concordance. Only isolated departures between Parmbsc0_εζOLI_ and CHARMM36 appear when the variation of the helical parameters is examined in individual complementary steps (examples in S9 Fig). In P-MDs, the rolls of TpA•TpA are systematically 5±2.5° larger than in C-MDs, for all backbone combinations; the twist of CpG•CpG and CpA•TpG in BII•BII is 6.5±1.5° higher in P-MDs than in C-MDs.

**Fig 9:**
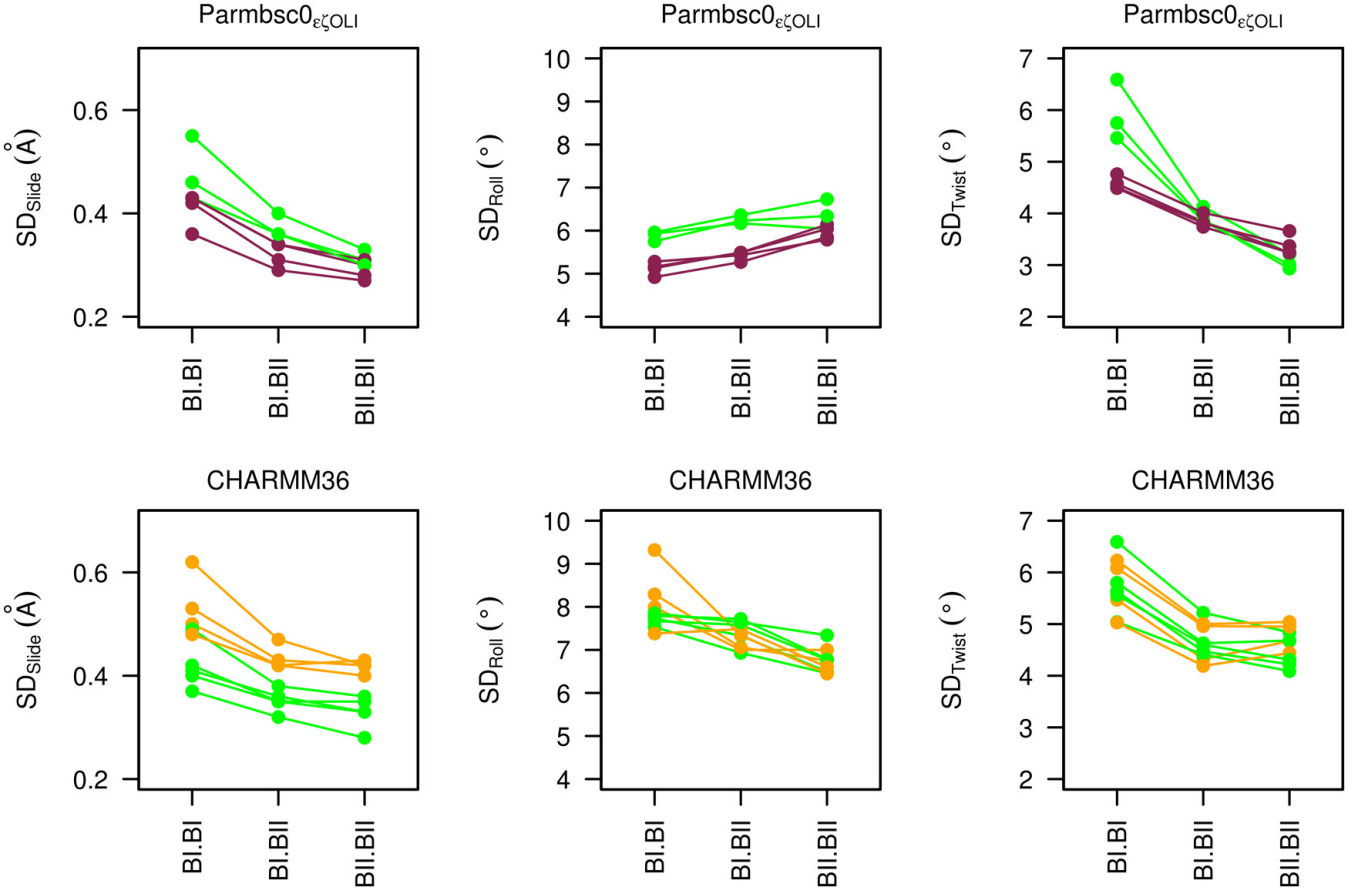
Variability of helical parameters depending on conformational combinations of facing phosphate linkages. The standard deviations of slide (SD_Slide_), roll (SD_Roll_) and twist (SD_Twist_) associated to the three possible combinations of facing phosphates are shown for representative steps, CpG•CpG in P-MDs and C-MDs of Oligos 1, 3 and 4 (green), GpC•GpC in P-MDs of Oligos 1, 2, 3 and 4 (violet) and TpA•TpA in C-MDs of Oligos 1, 3 and 4 (orange). Steps with the largest sampling of BII•BII (1.1 to 2.9%), were selected from P-MDs and C-MDs (7.5 to 21.8%). Top panels: P-MDs; bottom panels: C-MDs.

The MD results not only systematically documented the couplings, but they enabled comparison of the variability (standard deviations) of the helicoidal parameters for BI•BI, BI•BII|BII•BI and BII•BII. In both P-MDs and C-MDs, the slide and twist variabilities are greater in BI•BI compared to BI•BII|BII•BI or BII•BII (Fig 9). A similar, but attenuated, trend is observed for the roll in C-MDs (where the roll standard deviations are higher than in P-MDs). Thus, the simulations suggest that BII containing combinations are stiffer than BI•BI, at least for slide and twist. This, combined with the suppression of *north* sugars in 5’ of the BII linkages, might entropically disfavor the BII conformers. However, other contributions will affect the net balance of the BI ↔ BII equilibrium. Indeed, the above quantitative analysis makes clear that BII is frequently populated, and is the dominant conformer at some base steps.

The concordant results from P-MDs and C-MDs considerably strengthen and extend the view of the couplings between the facing phosphate states and inter base pair parameters gleaned from X-ray structures. Here, MDs inform about the behavior of a large range of dinucleotides, comprising those that are moderately or even barely propitious to BII. They reveal a general mechanical property of free DNA. Thus, BII containing complementary dinucleotides are characterized by more positive slide, more negative roll and higher twist than those in BI. As most steps have access to the BI•BII|BII•BI states (see the preceding section), the DNA deformation cost upon protein binding could be less important than expected when BII-like features are required for recognition. This can be illustrated by the TTAAA sequence in Oligo 3. This segment is considered as one of the strongest anchoring points in the nucleosome assembly [60–64], by forming multiple interactions with histones H3 and H4. In the X-ray structures of nucleosome containing the sequence 601 (PDB entries 3ZL0, 3ZL1 [63] and 3MVD [61]), TTAAA•TTTAA displays rather variable but globally negative rolls (-7 ±6°). According to both NMR and MDs, in their free state, these steps are mainly in BI•BI, associated to rolls of 4.4±3°. However, they also explore the BII•BI|BI•BII states, with rolls of -2.5±1°. So, the free TTAAA sequence spontaneously visits conformations closer than expected to its bound counterpart.

## Discussion

Assessing the extent to which MD simulations correctly represent B-DNA structural features in solution, their sequence dependency and populations remains an ongoing challenge and a necessary step to gain confidence in the role that DNA simulations may play in biophysics and structural biology. Part of the difficulty is to obtain experimental data in solution, suitable for comparison with simulations. Here, Parmbsc0_εζOLI_ [13] and CHARMM36 [11], specifically developed to improve the representation of the DNA backbone, were tested with respect to the sequence-specific BI and BII populations in four dodecamers, derived from ^31^P chemical shifts (δPs) [24].

The results show that the Parmbsc0_εζOLI_ and CHARMM36 potentials produce substantial BII populations, closer to their experimentally inferred counterpart than those obtained with preceding force fields [5, 9, 40, 41]. Many simulated BII propensities of the four dodecamers compare satisfactorily to experiment, a very encouraging achievement. This provides the foundation to understand the factors underpinning the differentiated BI ↔ BII equilibrium behavior across base steps. In particular, this context may be better adapted to investigate the quantum-mechanical origin of the phosphate chemical shifts [51].

However, the experimental sequence effect on BII propensities is still imperfectly reproduced by simulations, each force field displaying its own weaknesses. Parmbsc0_εζOLI_, as reported by its developers [13], globally underestimates the BII propensities. The CHARMM36 biases include generating too high BII percentages on TpA or, conversely, suppressing the BII character of GpC and some CpA and TpG. The procedure translating experimental δP to BII% is not devoid of uncertainties [51], but they would not account for the most severe discrepancies. For instance, the simulated TpA being BII-richer than GpC is clearly inconsistent with both NMR and X-ray data [15, 24, 28]. There was no evidence that the residual discrepancies in the sequence effect on the BI and BII populations resulted from insufficient sampling. The BII percentages were found converged well before the half microsecond timescale under monitored MD length increase. Since the BI↔BII exchange occurs in the pico to nanosecond time range in RMN experiments [21, 49], which is short compared to current simulation times, one does not expect that the BI↔BII equilibrium distributions would be significantly affected by increasing the sampling time. However, one cannot exclude the existence of hypothetical and currently unknown slower motions, on a longer time scale not probed by the present simulations, which might influence the BI ↔ BII equilibrium. Instead, progress is likely to require further refinements of the potentials. Considering the charged nature of the phosphate groups, it is possible that polarisable DNA force-fields will be required to reach a more satisfactory treatment of the sequence-dependent DNA properties [7, 65].

Despite some limitations, the present MDs are very helpful to scrutinize the DNA backbone dynamics, especially the behavior of facing phosphate groups within complementary dinucleotides. In that respect, Parmbsc0_εζOLI_ or CHARMM36 yielded similar features despite strong differences in their conception and parametrizations. This convergence strengthens the results. First the simulations indicate that concomitant BI ↔ BII transitions on two facing phosphates are much rarer than transitions involving only one phosphate. Second, statistical analysis of the simulations established that the conformational states of the two individual phosphates within a complementary dinucleotide were independent of each other. As a consequence, the BI•BI, BI•BII|BII•BI and BII•BII populations can be assessed from the individual BII percentages inferred from δPs, using straightforward equations. Importantly, this approach reveals that there is a sizable number of steps where BII-containing states dominate. Thus, more than one fourth of the 36 complementary dinucleotides spend more time in BI•BII|BII•BI and BII•BII than in BI•BI; all the complementary dinucleotides explore BI•BII|BII•BI in addition to BI•BI, with various populations of BI•BII|BII•BI; however, BII•BII is more restricted, apparently only significantly populated in a few types of BII-rich steps.

Since the behavior of facing phosphates was uncorrelated in all the 36 complementary steps studied here, one can reasonably infer that this is a general property of any B-DNA. Thus, according to the general and predictable sequence effect on experimental BII propensities [27, 28], the BII-containing combinations (BI•BII|BII•BI and BII•BII) are expected to be largely represented or even statistically dominant in CpG•CpG, CpA•TpG, GpC•GpC and GpG•CpC. The steps less propitious to BII, GpA•TpC, ApN•NpT (N: any base) and TpA•TpA, favor BI•BI but they also present modest fractions of BI•BII|BII•BI.

Such findings are of fundamental importance because of the strong couplings between these fine-grained backbone states and the inter base pair parameters of slide, roll and twist, consistent with initial observations on X-ray structures [15, 16]. Such couplings are not only confirmed here, but further characterized in solution for a broader range of steps, offering a unifying theme underpinning the intrinsic mechanics of B-DNA. Given the recurrent occurrence of BII-containing combinations, it follows that the accessible conformational landscape of most complementary dinucleotides extends into a region characterized by positive slide, negative roll and high twist (“BII profile”).

This enhanced intrinsic malleability is relevant to the reading of DNA by proteins, since it increases the repertoire of states which may be critical to initiate selective recognition by facilitating local, structural DNA adjustments upon protein binding. The implication of BII-rich steps in indirect readout mechanisms, *via* their ability to modulate the DNA shape, has been previously highlighted [9, 34, 43, 66]. In addition, the present work touched upon the counterintuitive example of the BI-rich (positive rolls) TTAAA segment in Oligo 3, which nevertheless also accesses negative rolls (BII•BI|BI•BII) in solution, reminiscent of the pattern of negative rolls observed in its nucleosome-bound form. So, the energetic penalty induced by the DNA deformation upon protein binding could be less than expected in many cases, especially when BII-like features are involved for the structural fit between the partners. Thus, the present characterization of free DNA is conceptually relevant to a deeper understanding of the selective recognition of DNA. The investigated force-fields Parmbsc0_εζOLI_ or CHARMM36 may also prove advantageous when simulating such events.

## Materials and Methods

### DNA sequences

Four oligodeoxyribonucleotides of 12 base pairs (bp) (sequences in Table 1) were studied by NMR and MD simulations. These sequences, placed end to end after discarding the terminal base pairs, recompose a continuous 39 bp segment corresponding to the 5’ part of the non-palindromic sequence 601, selected from SELEX experiments for its very high-affinity for association with the histone octamers [67].

### BII propensities from NMR

Sample preparation and NMR spectroscopy protocols were reported in a previous study [27]. All the NMR data are available in the Biological Magnetic Resonance Bank, entry 19222.

BII percentages (BII%) of the phosphate linkages along the four dodecamers were inferred from the phosphate chemical shifts (δPs, referenced to trimethyl phosphate) collected at 30°, using the equation BII(%) = 143 δP + 621 [24]. This equation is based on an empirical procedure that assumes the same δPs for purely BI or BII states of every dinucleotide, which is unlikely to be strictly correct [51]. Although previous studies showed that it is a reasonable approximation [27, 53], we decided to allow a large tolerance of ±10% on the BII percentages inferred from the experimental δPs to take into account uncertainty on the translation procedure.

### MDs with Parmbsc0_εζOLI_ and CHARMM36 force fields

MD simulations were performed with the Parmbsc0_εζOLI_ force-field [13] using the AMBER 14 program [68], or the CHARMM36 force-field [11] with program NAMD [69]. Parmbsc0_εζOLI_ and CHARMM36 simulations were carried out following protocols as comparable as possible. Yet, with Parmbsc0_εζOLI_ and CHARMM36, we used the counterion parameters classically associated to the Amber [70] and CHARMM [71] force-fields, respectively.

Parmbsc0_εζOLI_ and CHARMM36 simulations were performed at constant temperature (300K) and pressure (1bar) using the Berendsen algorithm [72]. The integration time-step was 2fs and covalent bonds involving hydrogen were constrained using SHAKE [73]. The non-bonded pair-list was updated heuristically. Long-range electrostatic interactions were treated using the particle mesh Ewald (PME) approach [74]. Non-bonded interactions were treated with a 9Å direct space cut-off in AMBER and with a force-shift function from 10 to 12 Å [75] with CHARMM36. In AMBER, the centre-of-mass motion was removed every 10ps.

With both Parmbsc0_εζOLI_ and CHARMM36, each dodecamer in initial standard B-DNA conformation was neutralized with 22 Na^+^ ions (minimal salt condition, ~50 mM Na^+^), in explicit TIP3P water molecules [76]; the primary boxes were truncated octahedrons with solvent extending 15Å around the DNA. The water molecules and counterions were energy-minimized and equilibrated at 100K around the constrained DNA for 100ps in the NVT ensemble; the entire system was then heated from 100 to 300K in 10ps by 5K increments with harmonic positional restraints of 5.0 kcal/mol/Å^2^ on the DNA atoms. The molecular dynamics simulations were continued in NPT, without notable change in volume. The positional restraints were gradually removed over 250ps and followed by the production phase. During the simulations, distance restraints were applied between base atoms of the first and last base pairs of each dodecamers, to prevent their opening. No restraint was applied on any of the internal nucleotides. The application of restraints on the terminal base pairs is justified in the next section, which highlights the benefits of conducting DNA simulations work alongside experimental characterization. MD snapshots were saved every 1 ps.

### Restrained base pairing on the first and last base pairs

During the simulations with Parmbsc0_εζOLI_ and CHARMM36, distance restraints were applied to maintain the Watson-Crick base-pairing in the first and last base pairs of each dodecamers, to prevent their opening. These restraints were applied on the terminal base pairs between base atoms involved in Watson-Crick hydrogen-bonding (Distance_donor/acceptor_ = 2.9±0.2Å) *via* a parabolic potential with a force-constant of 10 kcal/mol/Å^2^. The application of these restraints was motivated by the behavior of terminal base-pairs in unrestrained simulations, which are not presented in details here. We only give a summary of the unrestrained terminal base-pairs simulations compared to relevant NMR data, to justify the application of restraints in the presented MDs.

In the unrestrained simulations with Parmbsc0_εζOLI_, the first (N_1_:N_24_, N for any base type) and last (N_12_:N_13_) base pairs were generally open. These terminal bases, once extruded, got involved in various structural patterns that persisted during several hundreds of nanoseconds. In the most prevalent conformations, these bases interact with the penultimate phosphate group, insert into the minor groove or mispair with an antepenultimate base. These conformations impact some χ angles, are associated with unusual backbone dihedrals in N_1_pN_2_, N_11_pN_12_, N_13_pN_14_ or N_23_pN_24_, and break the stacking with the 3' or 5' neighbors (N_2_, N_11_, N_14_ or N_23_). With CHARMM36, the first two base pairs opened after a few nanoseconds and, as in Parmbsc0_εζOLI_ MDs, adopted multiple non-canonical structures. In these unrestrained MDs, base pair opening only occurred at the termini of the dodecamers and did not propagate further. Such behavior is not specific to our simulations since it was previously described for MDs with Parmbsc0 and Parmbsc0_OLI_ [5, 13, 48] or CHARMM36 [5], which used DNA sequences and simulation protocols different from the ones used here.

The re-orientation of the terminal bases towards the internal double stranded part of the DNA are not supported by the NMR data collected at 20 and 30°C on the four dodecamers. In one-dimensional ^1^H spectra at 30° (303K), the imino proton resonances are lost in the terminal base pairs while they are clearly observable in all the internal base pairs (from N_2_:N_23_ to N_11_:N_14_). This excludes long-lived disruption of the Watson-Crick hydrogen bonds in the penultimate base pairs (MDs with CHARMM36) or mispairing between a terminal base and an antepenultimate base (MDs with Parmbsc0_εζOLI_). The glycosidic bonds of the terminal nucleotides, probed by the intranucleotide distances H1'-H6/8, adopt the *anti* conformation. Furthermore, the numerous sequential NOEs between the penultimate and antepenultimate residues (N_2_pN_3_, N_10_pN_11_, N_14_pN_15_ or N_22_pN_23_) do not support extensive break of their stacking or abnormal structural features. NMR measurements also give information about the terminal steps, N_1_pN_2_, N_11_pN_12_, N_13_pN_14_ and N_23_pN_24_. The corresponding ^31^P chemical shifts are in the range of the internal phosphates. Intense, well defined ^31^P-^1^H4' couplings testify that the 3’ terminal phosphate groups conform to usual backbone conformation, since these couplings are observable only when α/β/γ are in *g-/trans/g+* [21, 77], the typical conformation of B-DNA. Finally, sequential NOE connectivities, clearly visible in all the terminal steps, imply that the fraying events do not generate large distance between open terminal bases and the penultimate residues.

In agreement with a detailed study of this topic [48], our NMR data indicate that current force fields do not yet provide a satisfactory description of the fraying of the terminal base pairs. The convergence issues induced by the behavior of the terminal regions in our unrestrained MDs are discussed in the Result section.

### Structural descriptors

The phosphate group linkages were characterized by torsion angles ε, ζ, α, β and γ following the conventional threefold staggered torsional pattern: gauche plus (60±40°), trans (180±40°) and gauche minus (300±40°). The sugar ring conformations were categorized according to their pseudorotation phase angle: *north* (300 to 50°), *east* (50 to 120°) and *south* (120 to 220°).

DNA structures were analyzed with Curves5 [78] and 3DNA [79]. Both programs produced almost identical helical parameter values. The inter base-pair parameters presented here for complementary dinucleotides NpN•NpN are those from Curves5. Only the 10 central base-pairs of each dodecamer were analyzed.

## Supporting Information

S1 Fig RMSD along the DNA trajectories with Parmbsc0_εζOLI_ and CHARMM36. (PDF)

S2 Fig Convergence of BII percentages in 450ns and 1 μs MD simulations. (PDF)

S3 Fig Convergence of BII percentages in 1 μs MD simulations of Oligo 4. (PDF)

S4 Fig Convergence of BII percentages in 1 μs P-MD simulations of Oligo 4, with or without restraints on the pairing of the first and last base pairs. (PDF)

S5 Fig (ε-ζ) distribution in X-ray structures. (PDF)

S6 Fig Influence of the force-field on the sugar puckers during the MD simulation of Oligo 4. (PDF)

S7 Fig Comparison between simulated and experimental BII percentages. (PDF)

S8 Fig Comparison between the conformational combinations of facing phosphate linkages extracted from C-MDs or based on NMR data. (PDF)

S9 Fig Slide, Roll and Twist values associated to conformational combinations of facing phosphate linkages in representative BII-rich steps, from C-MDs and P-MDs. (PDF)

S1 Table BII percentages from NMR and simulations with Parmbsc0_εζOLI_ and CHARMM36. (PDF)

S2 Table Populations of the three conformational combinations of facing phosphate groups based on experimental data. (PDF)

S3 Table Values of inter base pair parameters according to the conformational combinations of facing phosphate groups. (PDF)
