# Supplementary material for: Simulations Meet Experiment to Reveal New Insights into DNA Intrinsic Mechanics
Source: PLoS Comput Biol. 2015 Dec 10;11(12):e1004631. doi: 10.1371/journal.pcbi.1004631 (PMC4689557; doi:10.1371/journal.pcbi.1004631)
Supplement: S1 Fig — (PDF) [file pcbi.1004631.s001.pdf]

S1 Fig. RMSD along the trajectories with Parmbsc0$_{\varepsilon\zeta OLI}$ and CHARMM36.

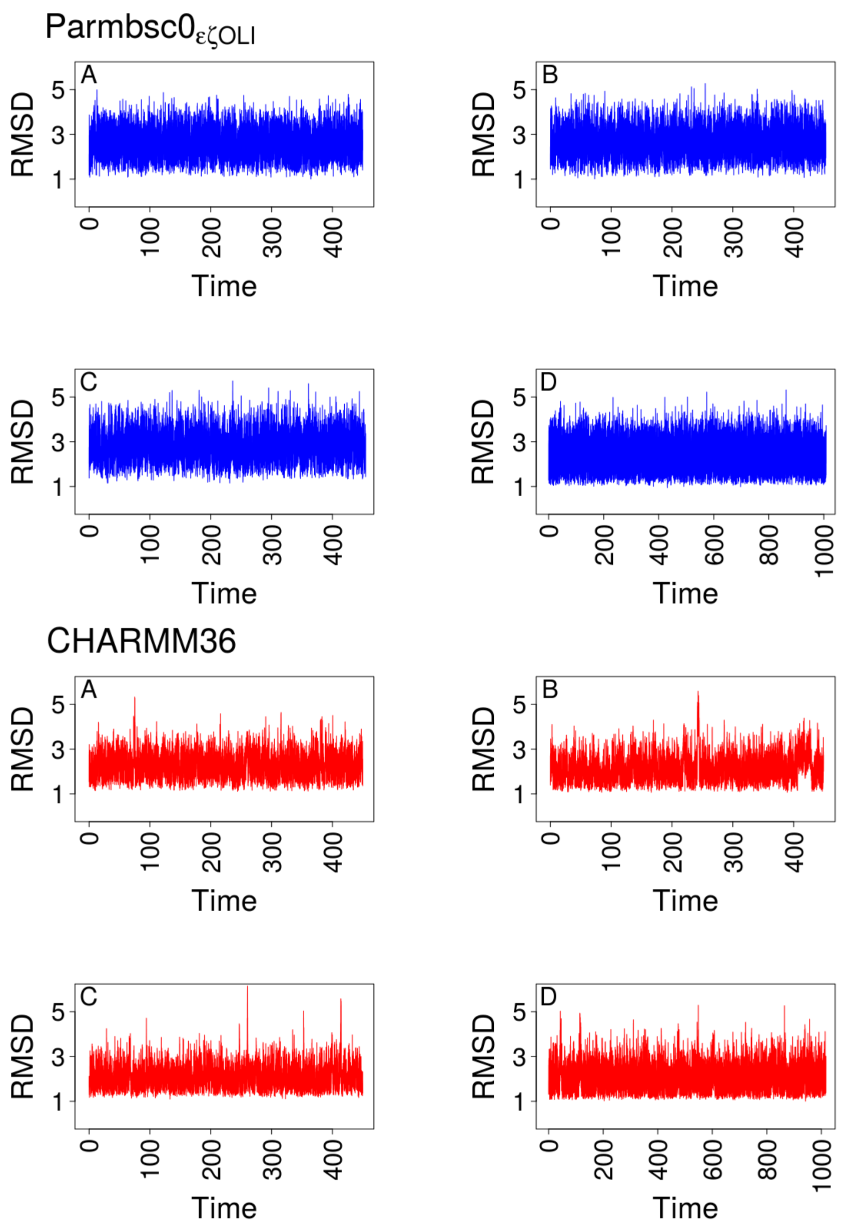

The Root Mean Square Deviations (RMSD, Å) are plotted in function of time (ns) for four DNA dodecamers (A: Oligo1; B: Oligo 2; C: Oligo 3; D: Oligo 4) simulated with either Parmbsc0$_{\varepsilon\zeta OLI}$ (top panels, in blue) or CHARMM36 (bottom panels, in red) force fields. RMSDs were calculated on the heavy atoms of the simulated structures -discarding the first and last base-pairs- and reference canonical B-DNAs generated by AMBER.

From: Simulations meet experiment to reveal new insights into DNA intrinsic mechanics

Akli Ben Imeddourene, Ahmad Elbahnsi, Marc Guéroult, Christophe Oguey, Nicolas Foloppe, and Brigitte Hartmann
