# Supplementary material for: Simulations Meet Experiment to Reveal New Insights into DNA Intrinsic Mechanics
Source: PLoS Comput Biol. 2015 Dec 10;11(12):e1004631. doi: 10.1371/journal.pcbi.1004631 (PMC4689557; doi:10.1371/journal.pcbi.1004631)
Supplement: S2 Fig — (PDF) [file pcbi.1004631.s002.pdf]

S2 Fig. Convergence of BII percentages in 450ns and 1μs MD simulations.

Oligo 1

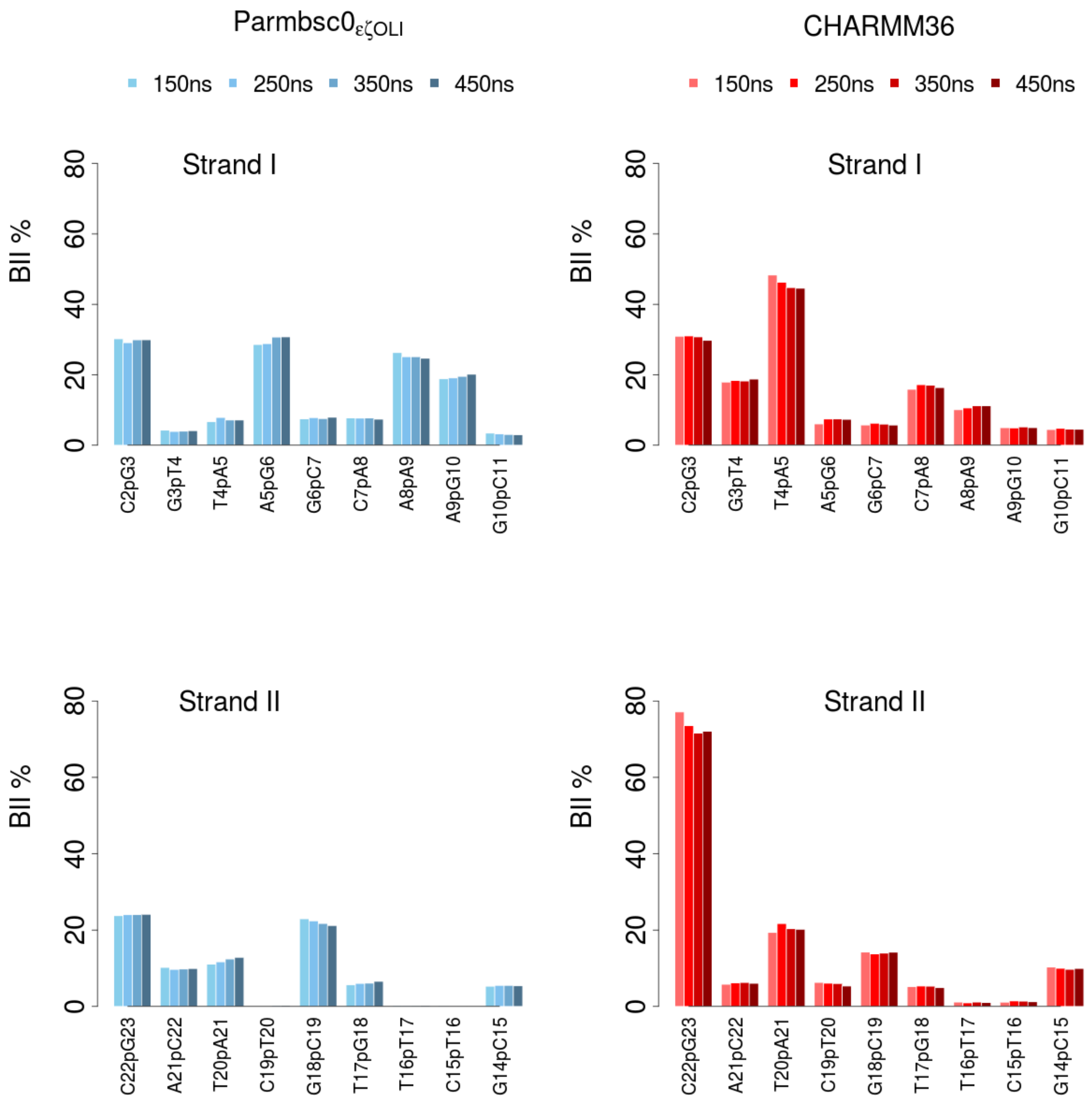

Cumulative BII percentages (BII%) of the dinucleotide steps were extracted from trajectories carried out with either Parmbsc0$_{\varepsilon\zeta OLI}$ (left, blue) or CHARMM36 (right, red) force fields. BII% were calculated for increasing length of MDs. The BII% were clearly not stabilized during the first 50ns, which are not considered in this analysis. To help identification of the facing steps, sequence in Strand 1 is shown from 5’ to 3’, while sequence in Strand 2 is shown from 3’ to 5’.

Oligo 2

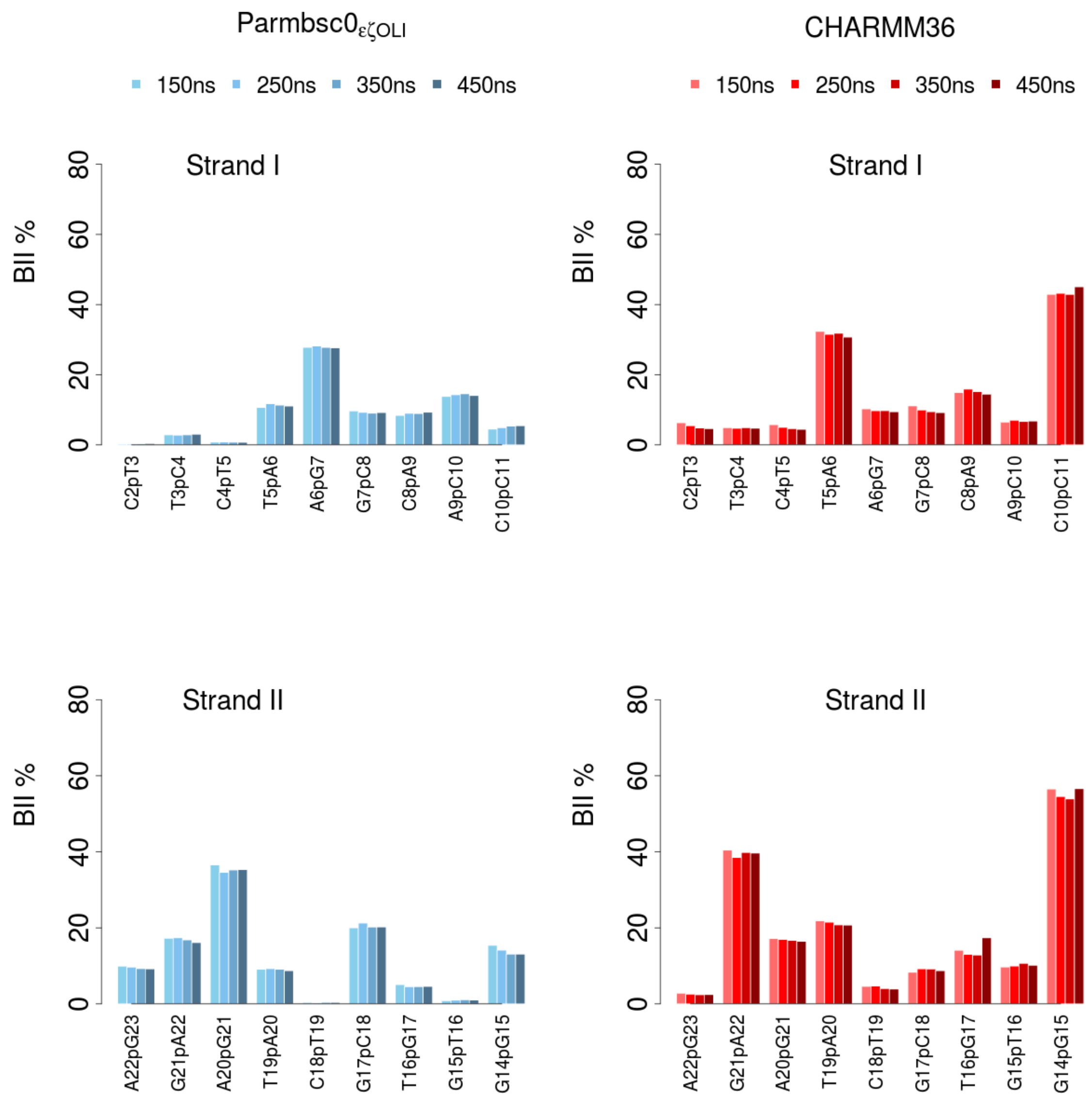

Cumulative BII percentages (BII%) of the dinucleotide steps were extracted from trajectories carried out with either Parmbsc0$_{\varepsilon\zeta OLI}$ (left, blue) or CHARMM36 (right, red) force fields. BII% were calculated for increasing length of MDs. The BII% were clearly not stabilized during the first 50ns, which are not considered in this analysis. To help identification of the facing steps, sequence in Strand 1 is shown from 5’ to 3’, while sequence in Strand 2 is shown from 3’ to 5’.

Oligo 3

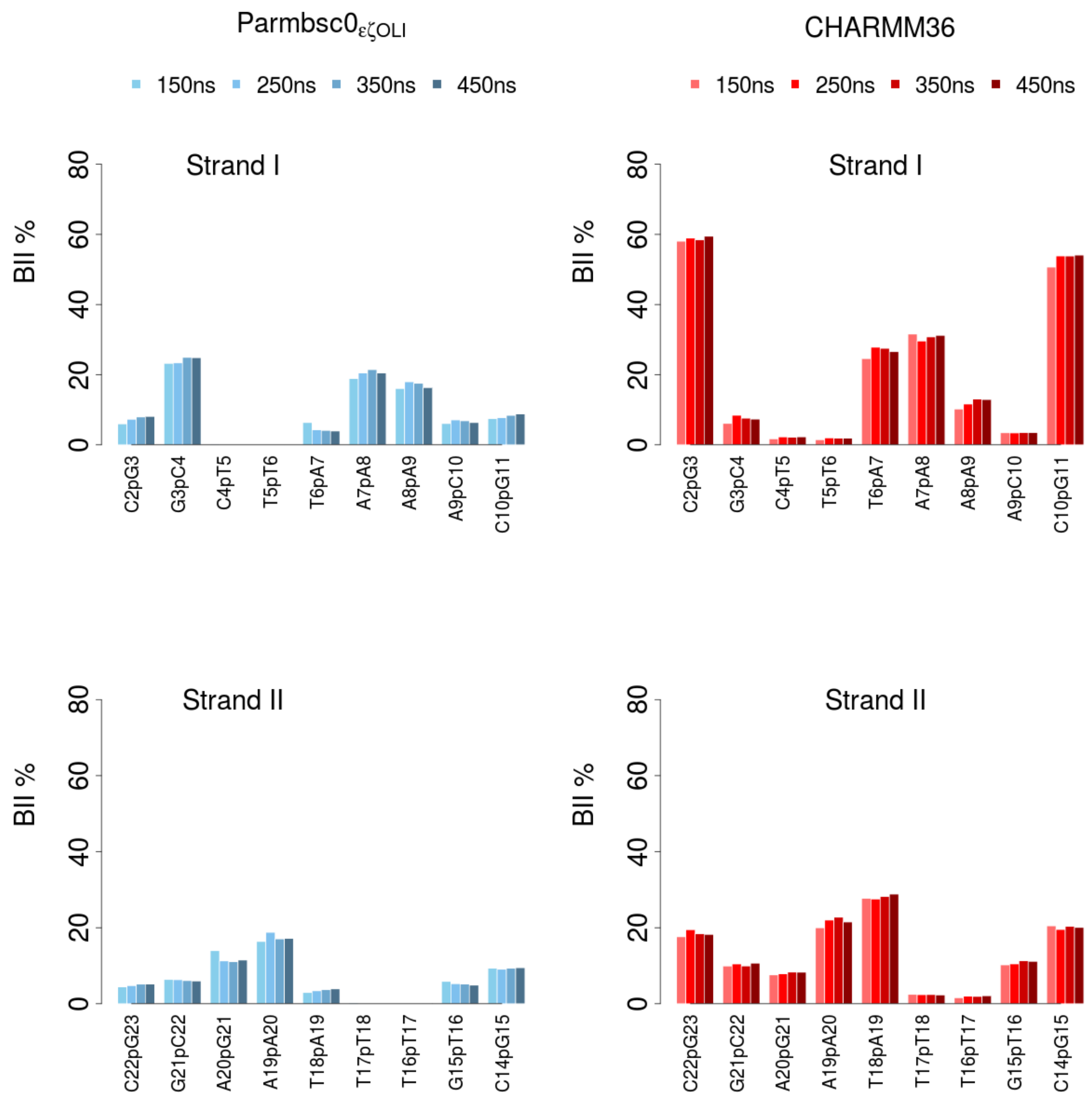

Cumulative BII percentages (BII%) of the dinucleotide steps were extracted from trajectories carried out with either Parmbsc0$_{\varepsilon\zeta OLI}$ (left, blue) or CHARMM36 (right, red) force fields. BII% were calculated for increasing length of MDs. The BII% were clearly not stabilized during the first 50ns, which are not considered in this analysis. To help identification of the facing steps, sequence in Strand 1 is shown from 5’ to 3’, while sequence in Strand 2 is shown from 3’ to 5’.

Oligo 4

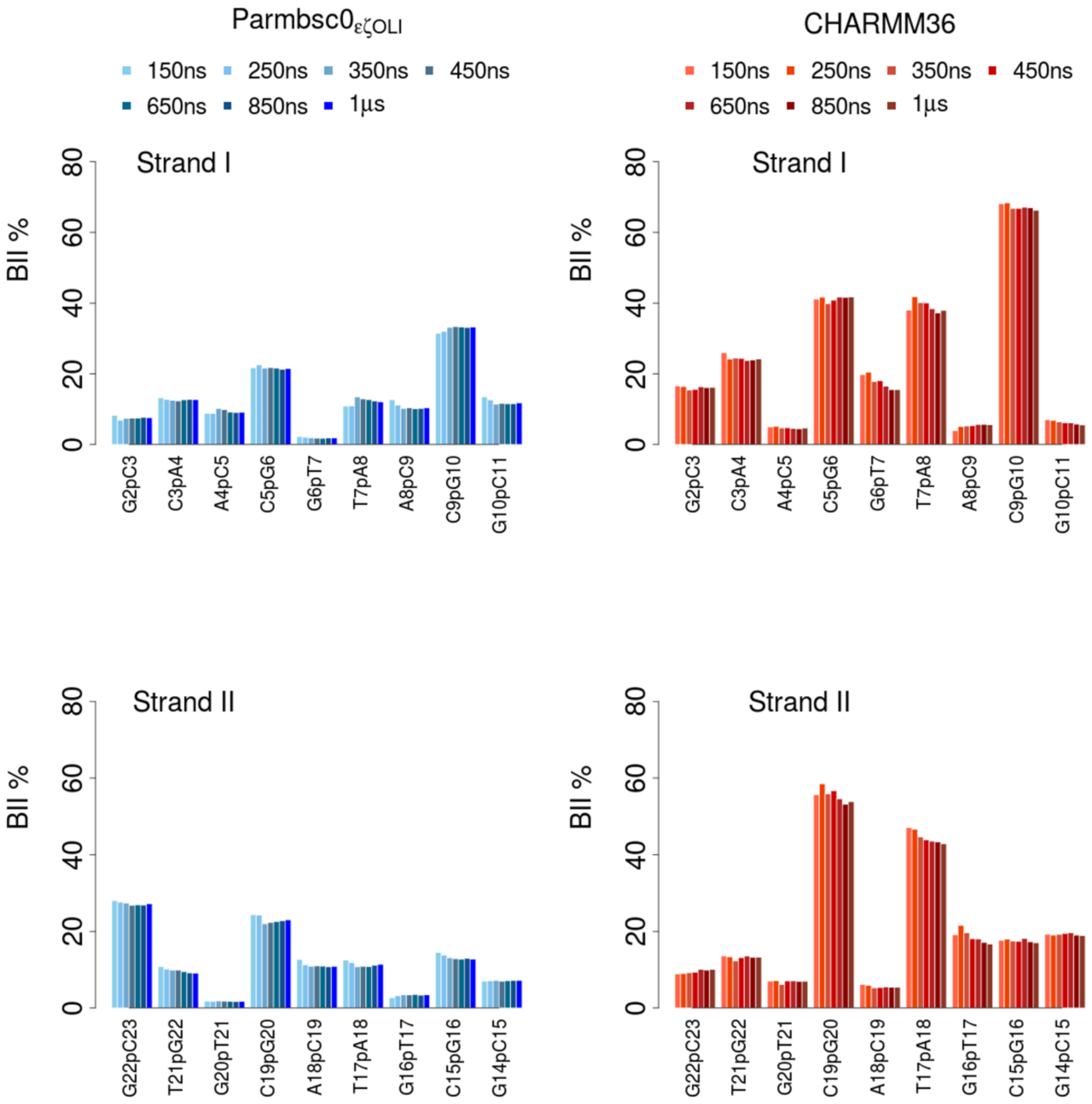

Cumulative BII percentages (BII%) of the dinucleotide steps were extracted from trajectories carried out with either Parmbsc0$_{\varepsilon\zeta OLI}$ (left, blue) or CHARMM36 (right, red) force fields. BII% were calculated for increasing length of MDs. The BII% were clearly not stabilized during the first 50ns, which are not considered in this analysis. To help identification of the facing steps, sequence in Strand 1 is shown from 5' to 3', while sequence in Strand 2 is shown from 3' to 5'.

From: Simulations meet experiment to reveal new insights into DNA intrinsic mechanics
Akli Ben Imeddourene, Ahmad Elbahnsi, Marc Guéroult, Christophe Oguey, Nicolas Foloppe, and Brigitte Hartmann
