# Supplementary material for: Simulations Meet Experiment to Reveal New Insights into DNA Intrinsic Mechanics
Source: PLoS Comput Biol. 2015 Dec 10;11(12):e1004631. doi: 10.1371/journal.pcbi.1004631 (PMC4689557; doi:10.1371/journal.pcbi.1004631)
Supplement: S3 Fig — (PDF) [file pcbi.1004631.s003.pdf]

S3 Fig. Convergence of BII percentages in 1µs MD simulations of Oligo 4.

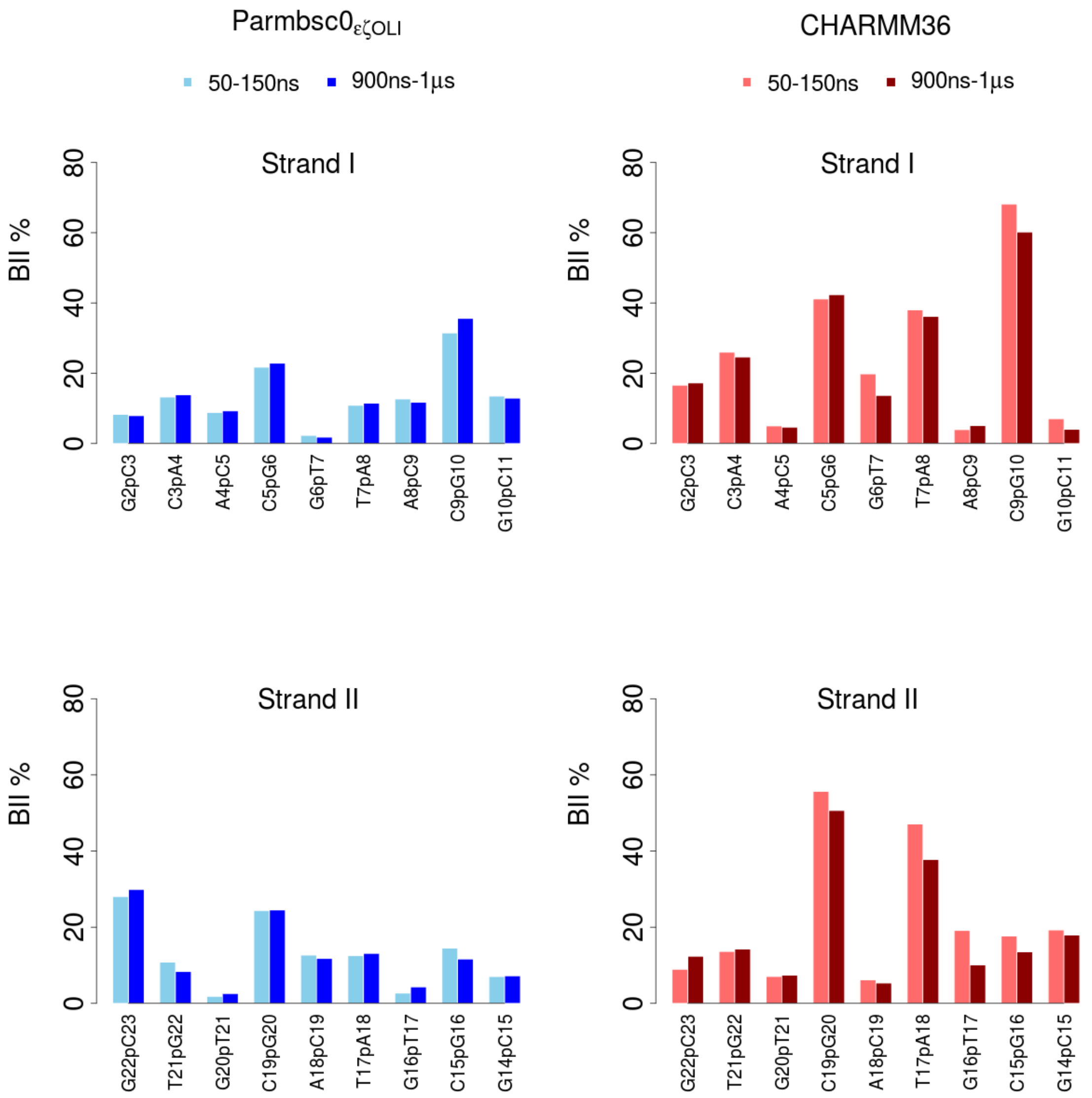

BII percentages (BII%) of the dinucleotide steps were extracted from trajectories carried out with either Parmbsc0$_{\varepsilon\zeta OLI}$ (left, blue) or CHARMM36 (right, red) force fields on Oligo 4. BII% were calculated for the beginning (-from 50 to 150ns, first bar) and the end (from 900 to 1000ns, second bar) of the trajectories, 100ns of MDs. The first 50ns are not considered in this analysis. To help identification of the facing steps, sequence in Strand 1 is shown from 5' to 3', while sequence in Strand 2 is shown from 3' to 5'.

From: Simulations meet experiment to reveal new insights into DNA intrinsic mechanics
Akli Ben Imeddourene, Ahmad Elbahnsi, Marc Guéroult, Christophe Oguey, Nicolas Foloppe, and Brigitte Hartmann
