# Supplementary material for: Simulations Meet Experiment to Reveal New Insights into DNA Intrinsic Mechanics
Source: PLoS Comput Biol. 2015 Dec 10;11(12):e1004631. doi: 10.1371/journal.pcbi.1004631 (PMC4689557; doi:10.1371/journal.pcbi.1004631)
Supplement: S4 Fig — (PDF) [file pcbi.1004631.s004.pdf]

S4 Fig. Convergence of BII percentages in 1µs P-MD simulations of Oligo 4, with or without restraints on the pairing of the first and last base pairs.

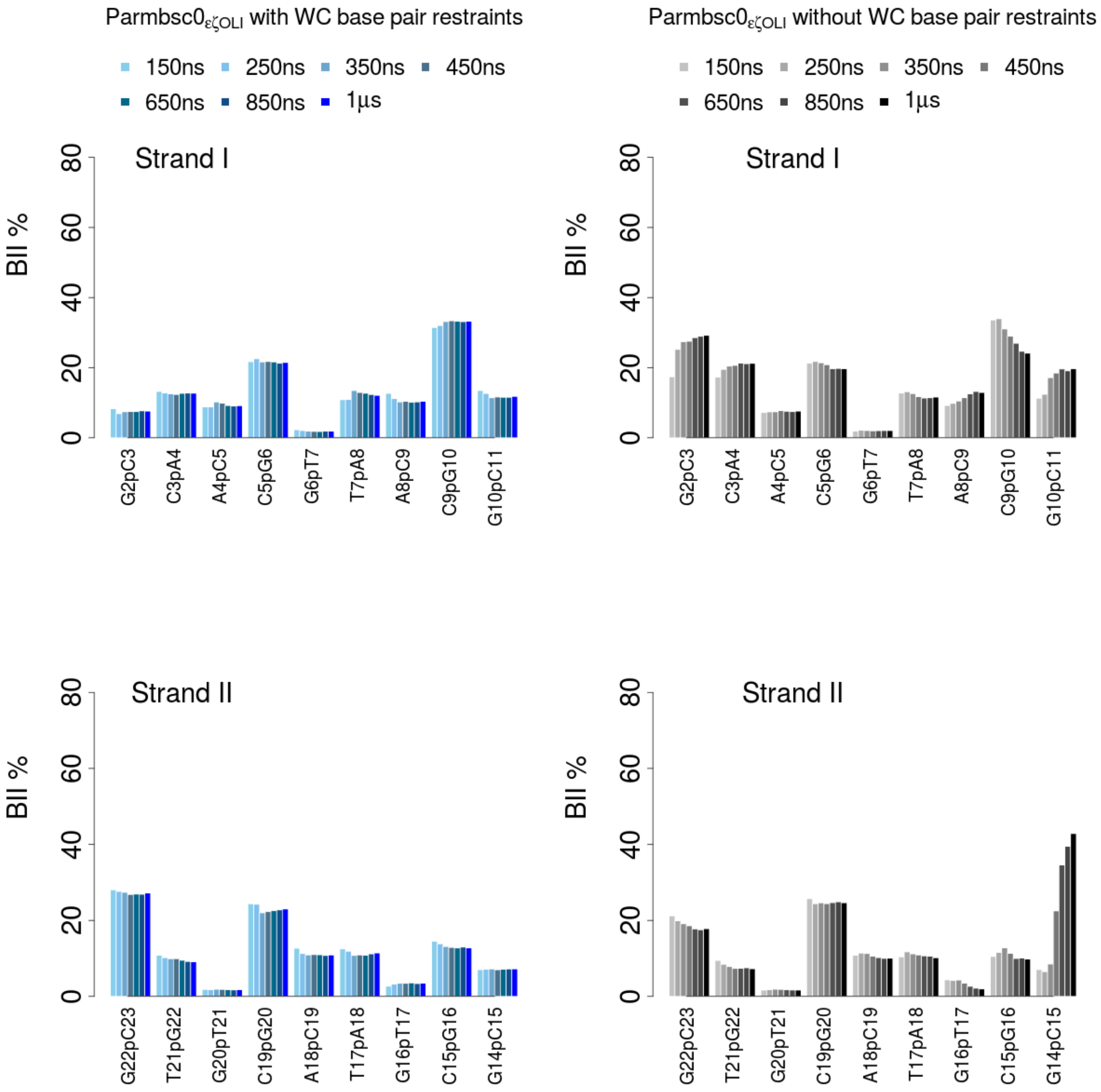

Cumulative BII percentages (BII%) of the dinucleotide steps were extracted from trajectories carried out with Parmbsc0$_{\varepsilon\zeta OLI}$ with (left, blue) or without (right, grey red) restraining the Watson-Crick pairing of the first and last base pairs. BII% were calculated for increasing length of MDs. The first 50ns are not considered in this analysis. To help identification of the facing steps, sequence in Strand 1 is shown from 5' to 3', while sequence in Strand 2 is shown from 3' to 5'. The convergence issues illustrated here with the unrestrained P-MD of Oligo 4 also occur with the three other Oligos and with the corresponding unrestrained C-MDs.

From: Simulations meet experiment to reveal new insights into DNA intrinsic mechanics
Akli Ben Imeddourene, Ahmad Elbahnsi, Marc Guéroult, Christophe Oguey, Nicolas Foloppe, and Brigitte Hartmann
