# Supplementary material for: Simulations Meet Experiment to Reveal New Insights into DNA Intrinsic Mechanics
Source: PLoS Comput Biol. 2015 Dec 10;11(12):e1004631. doi: 10.1371/journal.pcbi.1004631 (PMC4689557; doi:10.1371/journal.pcbi.1004631)
Supplement: S5 Fig — (PDF) [file pcbi.1004631.s005.pdf]

S5 Fig. (ε−ζ) distribution in X-ray structures.

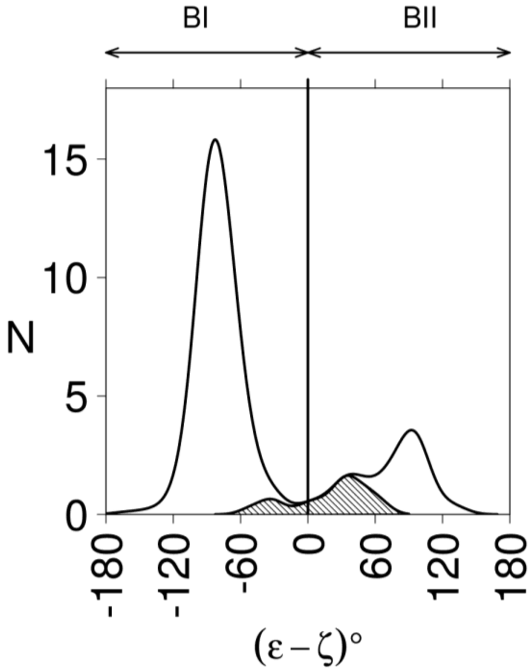

The frequencies (N) of (ε−ζ) values (°) were extracted from 431 steps of high resolution X-ray structures of free DNA. The hachured region corresponds to the distribution of ε/ζ:*trans/trans*.

From: Simulations meet experiment to reveal new insights into DNA intrinsic mechanics
Akli Ben Imeddourene, Ahmad Elbahnsi, Marc Guéroult, Christophe Oguey, Nicolas Foloppe, and Brigitte Hartmann
