# Supplementary material for: Simulations Meet Experiment to Reveal New Insights into DNA Intrinsic Mechanics
Source: PLoS Comput Biol. 2015 Dec 10;11(12):e1004631. doi: 10.1371/journal.pcbi.1004631 (PMC4689557; doi:10.1371/journal.pcbi.1004631)
Supplement: S6 Fig — (PDF) [file pcbi.1004631.s006.pdf]

S6 Fig. Influence of the force-field on the sugar puckers during the MD simulation of Oligo 4.

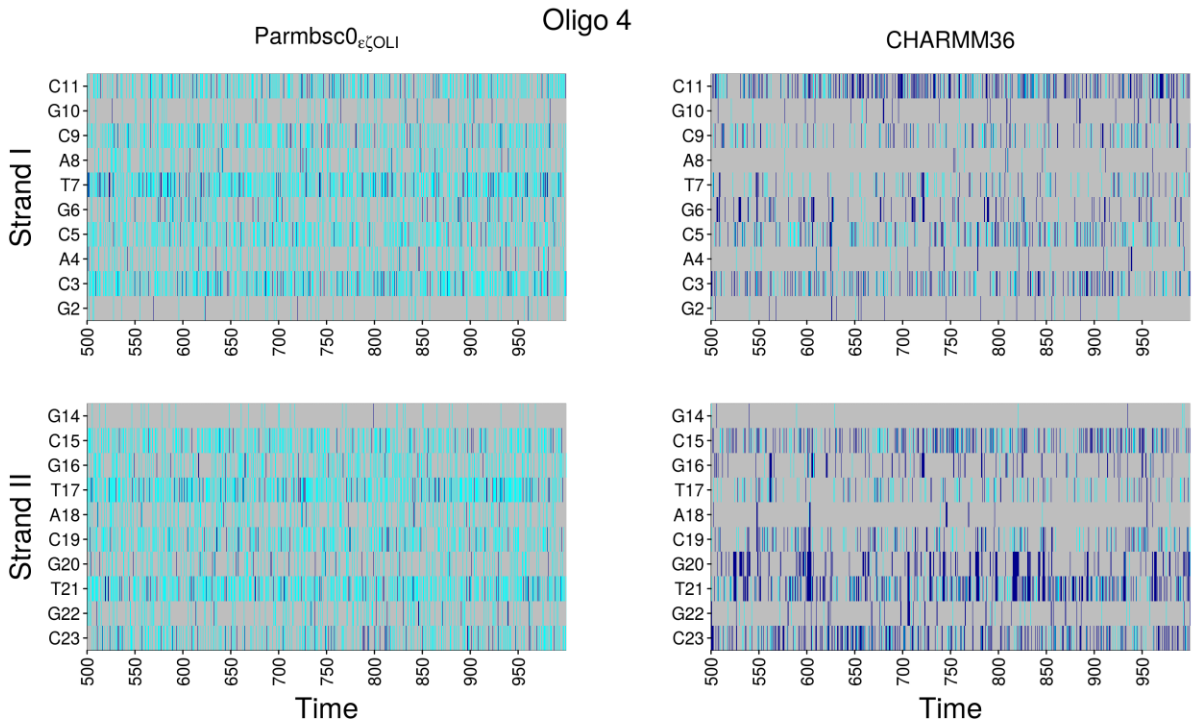

In MDs, the sugars interchange between three puckers, *south* (grey), *east* (cyan) and *north* (blue). The evolution of these conformations versus time (ns) is illustrated for the sugars along the two strands of Oligo 4 in the second part of µs MDs carried out with Parmbsc0$_{\varepsilon\zeta OLI}$ (left panels) or CHARMM36 (right panels). The sugar puckers are influenced by the force-field (see also Fig 2).

From: Simulations meet experiment to reveal new insights into DNA intrinsic mechanics
Akli Ben Imeddourene, Ahmad Elbahnsi, Marc Guéroult, Christophe Oguey, Nicolas Foloppe, and Brigitte Hartmann
