# Supplementary material for: Simulations Meet Experiment to Reveal New Insights into DNA Intrinsic Mechanics
Source: PLoS Comput Biol. 2015 Dec 10;11(12):e1004631. doi: 10.1371/journal.pcbi.1004631 (PMC4689557; doi:10.1371/journal.pcbi.1004631)
Supplement: S7 Fig — (PDF) [file pcbi.1004631.s007.pdf]

S7 Fig. Comparison between simulated and experimental BII percentages.

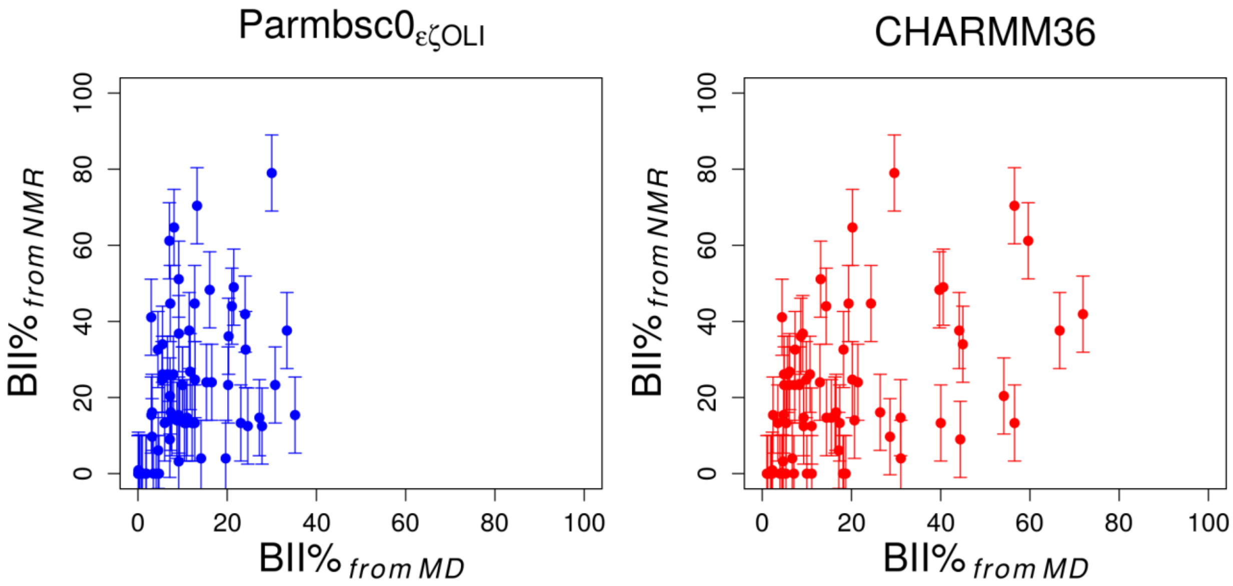

BII percentages were extracted from MDs (BII%$_{from\ MD}$) with Parmbsc0$_{\varepsilon\zeta OLI}$ (left panels, blue points) or CHARMM36 (right panels, red points) and compared with the corresponding values inferred from δPs (BII%$_{from\ NMR}$). The vertical bars represent the error on BII%$_{from\ NMR}$. The correlation coefficients are given in Table 2 (text section "Sequence-dependent BII propensities from simulations versus NMR.".

From: Simulations meet experiment to reveal new insights into DNA intrinsic mechanics
Akli Ben Imeddourene, Ahmad Elbahnsi, Marc Guéroult, Christophe Oguey, Nicolas Foloppe, and Brigitte Hartmann
