# Supplementary material for: Simulations Meet Experiment to Reveal New Insights into DNA Intrinsic Mechanics
Source: PLoS Comput Biol. 2015 Dec 10;11(12):e1004631. doi: 10.1371/journal.pcbi.1004631 (PMC4689557; doi:10.1371/journal.pcbi.1004631)
Supplement: S8 Fig — (PDF) [file pcbi.1004631.s008.pdf]

S8 Fig. Comparison between the conformational combinations of facing phosphate linkages extracted from C-MDs or based on NMR data.

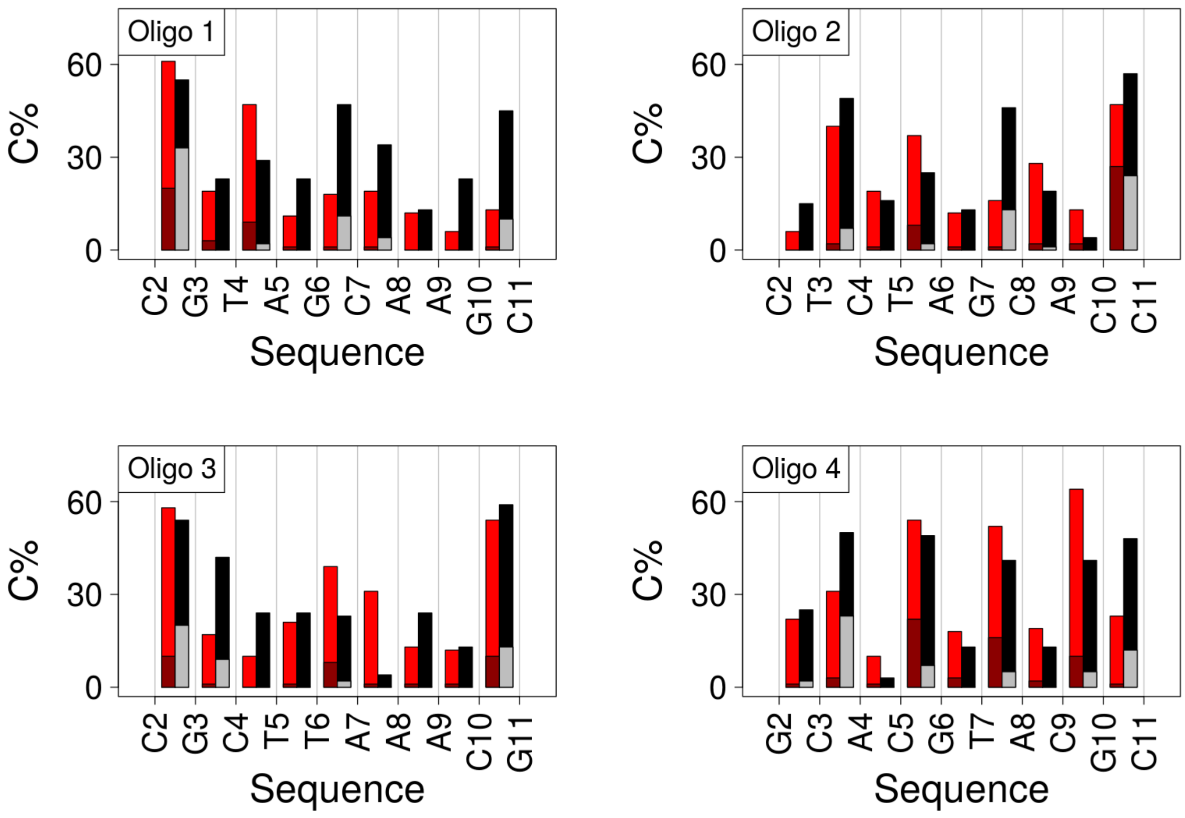

The percentages of BI•BII|BII•BI and BII•BII combinations (C%) of facing phosphates are plotted along the four dodecamer sequences. These percentages were extracted from C-MDs (red and dark red bars, stacked, for BI•BII|BII•BI and BII•BII, respectively). Here, they were compared with the percentages calculated in the regime of independence of the conformational states of facing phosphates with equations (2) and (3) (text section "New insights from the simulations: independence of the states of facing phosphate groups.") and the experimental data (black and grey bars, stacked, for BI•BII|BII•BI and BII•BII, respectively).

From: Simulations meet experiment to reveal new insights into DNA intrinsic mechanics
Akli Ben Imeddourene, Ahmad Elbahnsi, Marc Guéroult, Christophe Oguey, Nicolas Foloppe, and Brigitte Hartmann
