# Supplementary material for: Simulations Meet Experiment to Reveal New Insights into DNA Intrinsic Mechanics
Source: PLoS Comput Biol. 2015 Dec 10;11(12):e1004631. doi: 10.1371/journal.pcbi.1004631 (PMC4689557; doi:10.1371/journal.pcbi.1004631)
Supplement: S9 Fig — (PDF) [file pcbi.1004631.s009.pdf]

S9 Fig. Slide, Roll and Twist values associated to conformational combinations of facing phosphate linkages in representative BII-rich steps, generated by Parmbsc0$_{\varepsilon\zeta OLI}$ and CHARMM36.

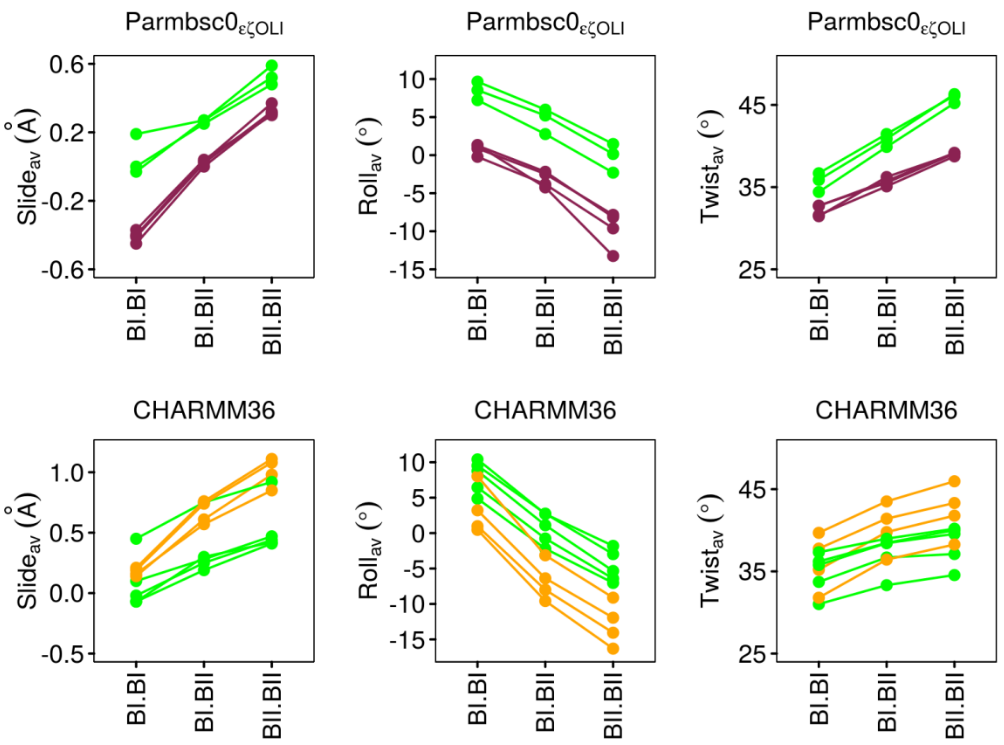

The values of Slide, Roll and Twist were calculated for representative BII-rich steps, CpG•CpG of Oligos 1, 3 and 4 (green), GpC•GpC of Oligos 1, 2, 3 and 4 (violet) and TpA•TpA of Oligos 1, 3 and 4 (orange) according to the BI•BI, BI•BII|BII•BI and BII•BII combinations of their facing phosphate groups. The data were extracted from C-MDs (top panels) and P-MDs (bottom panels), and averaged along the MD for each backbone state. For clarity, the standard deviations were omitted.

From: Simulations meet experiment to reveal new insights into DNA intrinsic mechanics
Akli Ben Imeddourene, Ahmad Elbahnsi, Marc Guéroult, Christophe Oguey, Nicolas Foloppe, and Brigitte Hartmann
