# Supplementary material for: Simulations Meet Experiment to Reveal New Insights into DNA Intrinsic Mechanics
Source: PLoS Comput Biol. 2015 Dec 10;11(12):e1004631. doi: 10.1371/journal.pcbi.1004631 (PMC4689557; doi:10.1371/journal.pcbi.1004631)
Supplement: S1 Table — (PDF) [file pcbi.1004631.s010.pdf]

S1 Table. BII percentages from NMR and simulations with Parmbsc0$_{\varepsilon\zeta OLI}$ and CHARMM36.

	Strand 1	BII%$_{from\ NMR}$	BII%$_{from\ P\text{-}MD}$	BII%$_{from\ C\text{-}MD}$	Strand 2	BII%$_{from\ NMR}$	BII%$_{from\ P\text{-}MD}$	BII%$_{from\ C\text{-}MD}$
**Oligo 1**	C2pG3	79	30	30	C22pG23	42	23	75
	G3pT4	0	4	19	A21pC22	23	17	6
	T4pA5	9	7	46	T20pA21	25	14	20
	A5pG6	23	31	7	C19pT20	0	3	6
	G6pC7	26	8	6	G18pC19	44	21	14
	C7pA8	16	7	16	T17pG18	26	10	5
	A8pA9	13	24	10	T16pT17	0	1	1
	A9pG10	23	20	5	C15pT16	0	1	1
	G10pC11	41	3	4	G14pC15	25	11	10
**Oligo 2**	C2pT3	0	0	6	A22pG23	15	14	2
	T3pC4	15	3	5	G21pA22	48	20	40
	C4pT5	0	1	5	A20pG21	15	38	17
	T5pA6	15	11	33	T19pA20	14	10	20
	A6pG7	13	28	9	C18pT19	0	0	4
	G7pC8	37	9	10	G17pC18	36	21	8
	C8pA9	15	9	14	T16pG17	6	10	13
	A9pC10	4	14	6	G15pT16	0	1	8
	C10pC11	34	6	43	G14pG15	70	43	57
**Oligo 3**	C2pG3	61	7	59	C22pG23	33	3	17
	G3pC4	33	24	7	G21pC22	26	5	11
	C4pT5	1	0	2	A20pG21	23	11	8
	T5pT6	0	0	2	A19pA20	24	21	20
	T6pA7	16	3	25	T18pA19	10	3	29
	A7pA8	4	20	31	T17pT18	0	0	3
	A8pA9	24	16	13	T16pT17	0	0	2
	A9pC10	13	6	4	G15pT16	0	4	11
	C10pG11	20	7	53	C14pG15	65	10	20
**Oligo 4**	G2pC3	15	8	17	G22pC23	15	24	9
	C3pA4	45	13	24	T21pG22	51	11	15
	A4pC5	3	9	6	G20pT21	0	2	8
	C5pG6	49	21	40	C19pG20	13	30	55
	G6pT7	0	2	19	A18pC19	13	12	6
	T7pA8	13	12	38	T17pA18	38	14	45
	A8pC9	13	10	5	G16pT17	0	5	19
	C9pG10	38	33	67	C15pG16	13	15	18
	G10pC11	27	12	7	G14pC15	45	7	18

The BII percentages of the individual phosphates were inferred from δPs (BII%$_{from\ NMR}$, with a tolerance of ±10%) or extracted from the simulations carried out with Parmbsc0$_{\varepsilon\zeta OLI}$ (BII%$_{from\ P\text{-}MD}$) or CHARMM36 (BII%$_{from\ C\text{-}MD}$).

From: Simulations meet experiment to reveal new insights into DNA intrinsic mechanics
Akli Ben Imeddourene, Ahmad Elbahnsi, Marc Guéroult, Christophe Oguey, Nicolas Foloppe, and Brigitte Hartmann
