# Supplementary material for: Simulations Meet Experiment to Reveal New Insights into DNA Intrinsic Mechanics
Source: PLoS Comput Biol. 2015 Dec 10;11(12):e1004631. doi: 10.1371/journal.pcbi.1004631 (PMC4689557; doi:10.1371/journal.pcbi.1004631)
Supplement: S2 Table — (PDF) [file pcbi.1004631.s011.pdf]

S2 Table. Populations of the three conformational combinations of facing phosphate groups based on experimental data.

		BI•BI%	BI•BII|BI•BII%	BII•BII%
**Oligo 1**	C2pG3•C22pG23	12	55	33
	G3pT4•A21pC22	77	23	0
	T4pA5•T20pA21	69	29	2
	A5pG6•C19pT20	77	23	0
	G6pC7•G18pC19	41	47	11
	C7pA8•T17pG18	62	34	4
	A8pA9•T16pT17	88	13	0
	A9pG10•C15pT16	77	23	0
	G10pC11•G14pC15	44	45	10
**Oligo 2**	C2pT3•A22pG23	85	15	0
	T3pC4•G21pA22	44	49	7
	C4pT5•A20pG21	84	16	0
	T5pA6•T19pA20	73	25	2
	A6pG7•C18pT19	88	13	0
	G7pC8•G17pC18	40	46	13
	C8pA9•T16pG17	80	19	1
	A9pC10•G15pT16	96	4	0
	C10pC11•G14pG15	20	57	24
**Oligo 3**	C2pG3•C22pG23	26	54	20
	G3pC4•G21pC22	49	42	9
	C4pT5•A20pG21	76	24	0
	T5pT6•A19pA20	76	24	0
	T6pA7•T18pA19	76	23	2
	A7pA8•T17pT18	96	4	0
	A8pA9•T16pT17	76	24	0
	A9pC10•G15pT16	87	13	0
	C10pG11•C14pG15	28	59	13
**Oligo 4**	G2pC3•G22pC23	73	25	2
	C3pA4•T21pG22	27	50	23
	A4pC5•G20pT21	97	3	0
	C5pG6•C19pG20	44	49	7
	G6pT7•A18pC19	87	13	0
	T7pA8•T17pA18	54	41	5
	A8pC9•G16pT17	87	13	0
	C9pG10•C15pG16	54	41	5

The percentages of the BII•BII, BI•BII|BII•BI and BII•BII combinations were calculated for the four dodecamers from the BII percentages of individual phosphates, BII%$_{from\ NMR}$, (see S1 Table) using the equations (2) and (3) given in the text section "New insights from the simulations: independence of the states of facing phosphate groups.".

From: Simulations meet experiment to reveal new insights into DNA intrinsic mechanics
Akli Ben Imeddourene, Ahmad Elbahnsi, Marc Guéroult, Christophe Oguey, Nicolas Foloppe, and Brigitte Hartmann
