# Supplementary material for: Simulations Meet Experiment to Reveal New Insights into DNA Intrinsic Mechanics
Source: PLoS Comput Biol. 2015 Dec 10;11(12):e1004631. doi: 10.1371/journal.pcbi.1004631 (PMC4689557; doi:10.1371/journal.pcbi.1004631)
Supplement: S3 Table — (PDF) [file pcbi.1004631.s012.pdf]

S3 Table. Values of inter base pair parameters according to the conformational combinations of facing phosphate groups.

	Shift		Tilt		Rise	
	P-MDs	C-MDs	P-MDs	C-MDs	P-MDs	C-MDs
BI•BI	0 (0.1)	0 (0.5)	-0.2 (1.5)	0 (5)	3.3 (0.1)	3.4 (0.4)
BI•BII|BII•BI	0 (0.5)	0 (0.6)	-0.4 (2.4)	-0.1 (5.5)	3.4 (0.1)	3.5 (0.4)
BII•BII	0 (0.2)	0 (0.5)	-0.5 (2.6)	-0.1 (5.7)	3.5 (0.2)	3.6 (0.5)

The characteristic values of Shift (Å), Tilt (°) and Rise (Å) with standard deviations in brackets are given for the three combinations of facing phosphate groups, regardless the dinucleotidic sequence. The inter base pair parameter values were extracted from the analysis of the simulations with Parmbsc0$_{\varepsilon\zeta OLI}$ (P-MDs) and CHARMM36 (C-MDs).

From: Simulations meet experiment to reveal new insights into DNA intrinsic mechanics
Akli Ben Imeddourene, Ahmad Elbahnsi, Marc Guéroult, Christophe Oguey, Nicolas Foloppe, and Brigitte Hartmann
